# Expression of receptor activator of NFkB (RANK) drives stemness and resistance to therapy in ER+HER2- breast cancer

**DOI:** 10.18632/oncotarget.27576

**Published:** 2020-05-12

**Authors:** Inês Gomes, Bernardo P. de Almeida, Sara Dâmaso, André Mansinho, Inês Correia, Sara Henriques, Raquel Cruz-Duarte, Guilherme Vilhais, Pedro Félix, Patrícia Alves, Patrícia Corredeira, Nuno L. Barbosa-Morais, Luis Costa, Sandra Casimiro

**Affiliations:** ^1^Luis Costa Laboratory, Instituto de Medicina Molecular, Faculdade de Medicina da Universidade de Lisboa, Lisboa, Portugal; ^2^Nuno Morais Laboratory, Instituto de Medicina Molecular, Faculdade de Medicina da Universidade de Lisboa, Lisboa, Portugal; ^3^Serviço de Oncologia, Hospital de Santa Maria-CHULN, Lisboa, Portugal; ^4^Current affiliation: Research Institute of Molecular Pathology (IMP), Vienna Biocenter (VBC), Vienna, Austria

**Keywords:** RANKL-RANK pathway, ER+ breast cancer, resistance to chemo and hormone therapy, stemness, metastization

## Abstract

The role of RANKL-RANK pathway in progesterone-driven mammary carcinogenesis and triple negative breast cancer tumorigenesis has been well characterized. However, and despite evidences of the existence of RANK-positive hormone receptor (HR)-positive breast tumors, the implication of RANK expression in HR-positive breast cancers has not been addressed before. Here, we report that RANK pathway affects the expression of cell cycle regulators and decreases sensitivity to fulvestrant of estrogen receptor (ER)-positive (ER+)/HER2- breast cancer cells, MCF-7 and T47D. Moreover, RANK overexpressing cells had a staminal and mesenchymal phenotype, with decreased proliferation rate and decreased susceptibility to chemotherapy, but were more invasive *in vivo*. *In silico* analysis of the transcriptome of human breast tumors, confirmed the association between *RANK* expression and stem cell and mesenchymal markers in ER+HER2- tumors. Importantly, exposure of ER+HER2- cells to continuous RANK pathway activation by exogenous RANKL, *in vitro* and *in vivo*, induced a negative feedback effect, independent of RANK levels, leading to the downregulation of HR and increased resistance to hormone therapy. These results suggest that ER+HER2- RANK-positive cells may constitute an important reservoir of slow cycling, therapy-resistance cancer cells; and that RANK pathway activation is deleterious in all ER+HER2- breast cancer cells, independently of RANK levels.

## INTRODUCTION

Breast cancer is responsible for over 600,000 deaths per year worldwide [1], the vast majority due to cancer spread into distant organs. Although hormone receptor (HR)-positive breast cancer has the better prognosis, 30% of cases will develop metastatic disease due to intrinsic or acquired resistance to therapy [2, 3].

The receptor activator of nuclear factor-kB ligand (RANKL)-RANK pathway was first identified as mediator of T and dendritic cells interaction [4], but it is mostly known for its role as key regulator of bone remodeling [5] and pathophysiology of bone metastases [6]. RANK is a member of the tumor necrosis factor receptor (TNFR) superfamily which is activated upon RANKL binding, promoting cell proliferation, survival and differentiation [7, 8].

The RANKL-RANK pathway also emerged as a major mediator of hormone-driven breast carcinogenesis. RANKL is expressed downstream of activated progesterone receptor (PR) in HR-positive mammary epithelial cells (MECs), and drives the proliferation of HR-negative RANK-expressing mammary stem cells (MaSCs) in a paracrine manner [9–12]. Overexpression of RANK in normal HR-negative MCF10A mammary cells induces stemness and transformation features, namely mammary gland reconstitution, epithelial-mesenchymal transition (EMT), increased migration, and anchorage-independent growth [13]; interfering with mammary cell commitment and contributing to breast carcinogenesis [14]. Furthermore, RANKL-RANK pathway enhances tumorigenesis and metastasis in BRCA1-driven breast cancer [13, 15, 16]. Therefore, the pharmacological blockade of RANKL-RANK pathway may control the incidence and onset of progestin-driven breast cancer and expansion of stem-cell-enriched populations.

In clinical samples, RANK was found to be expressed by different solid tumors [17], and RANK expression emerged as a predictive marker of breast cancer bone metastasis occurrence and shorter disease-free survival (DFS), being correlated with high grade and negative HR status [13, 18, 19]. However, it has been reported the existence of RANK-positive estrogen receptor (ER)-positive (ER+) breast cancers [19]. In preclinical studies, it was shown that RANKL triggers migration of RANK-positive human epithelial cancer cells [20]; and RANK overexpression in triple negative breast cancer (TNBC) cells was sufficient to confer a significantly greater metastatic growth rate in the bone, by inducing the expression of matrix metalloproteinases and other genes previously defined as part of a bone metastasis gene signature [21, 22].

Despite evidences of heterogeneous RANK expression amongst breast tumors, the implication of RANK expression in HR-positive breast cancers have remained elusive. Here, we report that ER+HER2- RANK-overexpressing breast cancer cells have a staminal and mesenchymal phenotype, with decreased proliferation rate and decreased susceptibility to chemotherapy and fulvestrant. Moreover, continuous RANK pathway activation in ER+HER2- cells induces a negative feedback effect, independent of RANK levels, leading to the downregulation of HR and increased resistance to hormone therapy (HT). Therefore, RANKL-RANK pathway may affect the outcomes of ER+HER2- breast cancer.

## RESULTS

### RANK overexpression in ER+HER2- breast cancer cell lines affects the expression of cell cycle regulators and decreases sensitivity to fulvestrant

**Figure 1:**
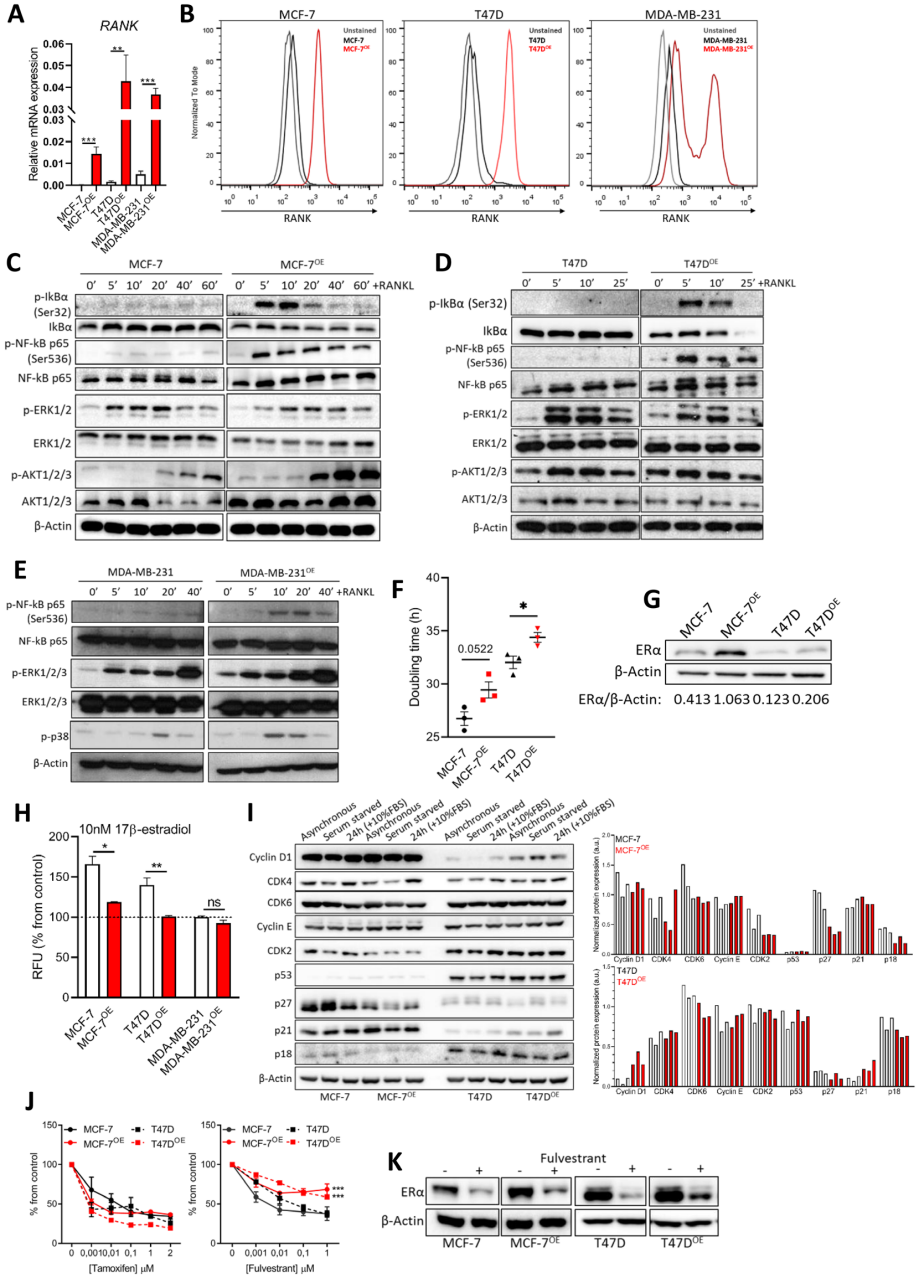
RANK overexpression is associated with altered cell cycle regulators and induced resistance to fulvestrant. (**A**) RT-qPCR of *RANK* in parental and RANK OE cell lines (*n* = 3). (**B**) Flow cytometry of RANK in parental and RANK OE cell lines. (**C**, **D**, **E**) Downstream targets of RANK were analyzed by western blot upon stimulus with 1 μg/ml RANKL for the indicated time points. β-Actin was used as loading control. (**F**) Doubling time was quantified under standard conditions, and calculated using exponential growth equation with least squares regression fitting model (*n* = 3). (**G**) Western blot of ER with β-Actin as loading control. (**H**) Cell viability was measured after 5 days of culture in steroids-depleted medium +/– 10 nM β-estradiol (*n* = 3). (**I**) Western blot analysis of cell cycle-related proteins with β-Actin as loading control. (**J**) Cell viability was measured 7 days after exposure to tamoxifen or fulvestrant, with medium replacement every 48 h. (*n* = 3). (**K**) Representative western blot of down-stream target of fulvestrant (ER) with β-Actin as loading control (*n* = 3). FiJi was used to obtain the best contrast for western blot band visualization, and background was removed for band densitometry analysis. Results are presented as the mean ± SEM. ^*^ *p* < 0.05, ^**^ *p* < 0.01, ^***^ *p* < 0.001.

In this work we proposed to investigate the effect of RANK expression in ER+HER2- breast cancer. To address this, we overexpressed RANK in two different ER+HER2- breast cancer cell lines, MCF-7 and T47D, which express low endogenous levels of this gene (Supplementary Table 1). MDA-MB-231 TNBC cells, known to express functional RANK [20, 22], and their RANK overexpressing (RANK OE) counterparts were used as comparators. We confirmed RANK OE by RT-qPCR and flow cytometry (Figure 1A, 1B); and RANK OE functionality by analyzing RANK pathway activation upon serum starvation and stimuli with exogenous RANKL (Figure 1C–1E). RANK pathway was hyper activated in luminal RANK OE cells, as shown by IkBα, NF-kB, ERK and AKT phosphorylation, and IkBα degradation. Since RANK-downstream effectors were also activated in MCF-7 and T47D parental cells, which express low levels of RANK, we next confirmed that protein phosphorylation was RANK-dependent by analyzing p65 phosphorylation in MCF-7 parental and RANK OE cells, in the presence of RANKL neutralized or not with MAB626, a RANKL-specific antibody (Supplementary Figure 1A). MAB626 was able to block p65 phosphorylation, confirming a RANK-dependent activation of downstream effectors by RANKL. We also observed pathway activation without RANKL in RANK OE cells (T0), suggesting that autocrine activation of RANK pathway may occur. Therefore we analyzed RANKL expression. RANKL was expressed in all cell lines (Supplementary Figure 1B, 1C), although not up-regulated in RANK OE cells. This was corroborated by quantification of sRANKL in conditioned culture media of luminal cells by ELISA (Supplementary Figure 1D).

Exposure to exogenous RANKL had no effect on luminal cells’ proliferation; however, RANK OE cells were less proliferative upon release from serum starvation in comparison with parental counterparts (Supplementary Figure 1E). We therefore quantified each cell line’s doubling time, which was higher in RANK OE cells (Figure 1F).

Since proliferation rate was negatively affected, we questioned if RANK OE impacts the expression of ER, a major regulator of proliferation in ER+ cells. We analyzed ER levels by western blot, and found ER to be up-regulated in RANK OE cell lines, although to a higher extent in MCF-7 cells (Figure 1G). However, upon estradiol deprivation RANK OE cells were significantly less sensitive to estradiol (Figure 1H). This may contribute to the decreased growth rate, and suggests that alternative pathways are involved in survival. To assess if RANK OE effects other proteins involved in cell cycle regulation, we synchronized cells in G0-G1 by serum starvation, followed by serum starvation-release with 10%FBS for 24 h (Supplementary Figure 1F). Comparison of MCF-7 and MCF-7OE cells shows a decrease in CDK2, p27 and p18 in RANK OE cells (Figure 1I). Moreover, serum starvation for 24 h had a very discrete effect in MCF-7OE cells. Comparison of T47D and T47DOE cells shows an increase in cyclinD1 and p21, and down-regulation of p27 and p18, in RANK OE cells. Again, serum starvation for 24 h had a very discrete effect in T47DOE cells, in opposite to T47D cells. This suggests the existence of compensatory mechanisms in RANK OE cells to sustain proliferation in stress conditions.

Because RANK OE cells were characterized by increased expression of ER but decreased sensitivity to estradiol, we questioned if this would affect the response to HT, standard of care for ER+ breast cancers in all settings. Drug sensitivity assays demonstrate that RANK OE cells had decreased sensitivity to fulvestrant but not to tamoxifen (Figure 1J). Tamoxifen is a selective estrogen receptor modulator (SERM), an agonist that allows partial activation of ER. Fulvestrant is, however, is a selective estrogen receptor down-regulator (SERD), a pure antagonist which competitively binds to ER and, in contrast to tamoxifen, induces a rapid degradation and loss of the ER protein. Since fulvestrant induces ER degradation in a dose dependent manner [23] and RANK OE cells overexpress the receptor, we hypothesized that fulvestrant was less effective due to sustained ER expression upon treatment. We confirmed our hypothesis by measuring ER in fulvestrant-treated cells, which was maintained at higher levels in comparison to parental cells (Figure 1K). This reinforces that other pathways are involved in survival of RANK+ ER+ breast cancer cells.

### RANK overexpression in ER+HER2- breast cancer cell lines induces mesenchymal and staminal characteristics

**Figure 2:**
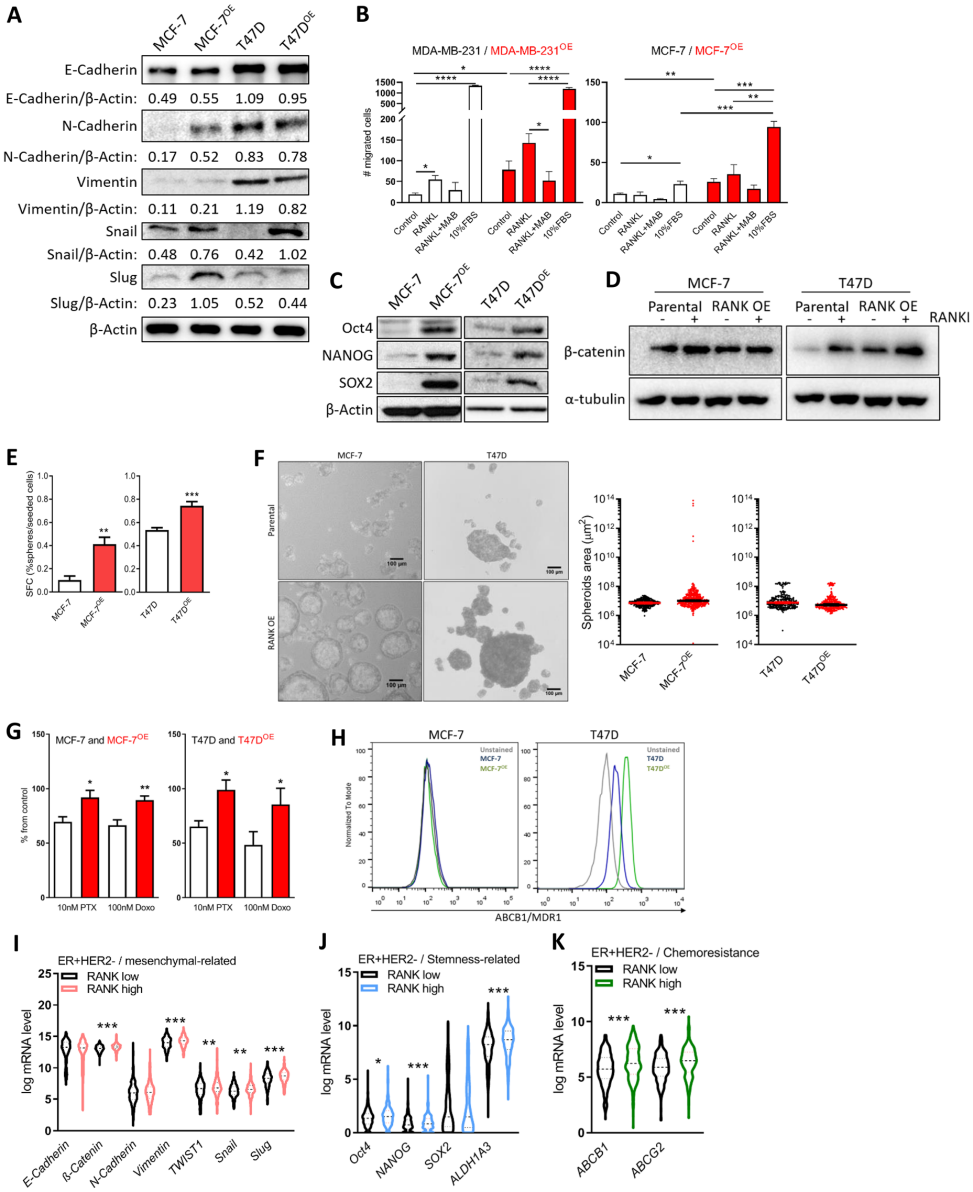
RANK OE cells exhibit mesenchymal and stem-cell like characteristics. (**A**) Expression of epithelial-mesenchymal markers was analyzed by western blot and β-Actin was used as loading control. (**B**) Cell migration was quantified after 24 h of stimuli with 2.5 μg/ml RANKL (+/-MAB626) or 10%FBS. (**C**) Expression of stemness-related markers was analyzed by western blot and β-Actin was used as loading control. (**D**) β-catenin was analyzed by western blot upon stimulus with 1 μg/ml RANKL for 90 min. β-Actin was used as loading control. (**E**) Sphere Forming Capacity (SFC) quantification as the number of tumorspheres > 50 μm/number of cells seeded × 100, after 7 days in non-adherent conditions. (*n* = 3) (**F**) Spheroids area with median and 95% CI considering all tumorspheres > 50 μm in diameter. (**G**) Cell viability was measured 72 h after exposure to paclitaxel (PTX) and doxorubicin (Doxo). (*n* = 3). (**H**) Flow cytometry analysis of MDR1 expression in MCF-7 and T47D cells (*n* = 2). (**I**–**K**) Interest gene expression in the ER+HER2- TCGA cohort (*n* = 587) according to *RANK* expression. FiJi was used to obtain the best contrast for western blot band visualization, and background was removed for band densitometry analysis. Results are presented as the mean ± SEM. ^*^ *p* < 0.05, ^**^ *p* < 0.01, ^***^ *p* < 0.001.

Next, and based on the reported effects of RANK OE in TNBC, we analyzed the expression of known epithelial and mesenchymal cell markers and observed an increase in Snail in both RANK OE cell lines (Figure 2A); whereas increased expression of N-cadherin, Vimentin and Slug was observed in MCF-7^OE^ cells, but not in T47D^OE^. We hypothesize that this can be due to the fact that T47D cells exhibit a more mesenchymal phenotype in comparison with MCF-7, with basal increased levels of these proteins.

Since increase in migration has been associated with RANK-pathway activation, we next assessed migration in the presence of 2.5 μg/ml RANKL, which was previously shown to induce cancer cells migration (Figure 2B and Supplementary Figure 2A). MCF-7 RANK OE cells were more invasive *per se*, and stimuli with RANKL increased migration, although not significantly. Like in MDA-MB-231 cells, RANKL effect was abrogated by RANKL neutralization with an anti-RANKL specific antibody.

Luminal RANK OE cells were also characterized by stem cell-like features, including Oct4, NANOG and SOX2 up-regulation (Figure 2C); β-catenin up-regulation (Figure 2D); and increased Sphere Forming Capacity (SFC; Figure 2E). We did not find a significant difference in the average tumorspheres’ area, although MCF-7 RANK OE-derived spheres were enriched in a sub-population of enlarged spheroids (Figure 2F). Exposure to RANKL did not increase the size of tumorspheres (Supplementary Figure 2B), but a more invasive phenotype was suggested by 3D culture (Supplementary Figure 2C).

Since chemoresistance is a hallmark of cancer stem cells, we next investigated the sensitivity of RANK OE cells to paclitaxel (PTX) and doxorubicin (Doxo), two chemotherapeutic agents commonly used to treat patients with breast cancer. RANK OE cells showed decreased sensitivity to both drugs, in comparison with parental cell lines (Figure 2G). Chemoresistance is often associated with elevated expression of drug efflux pumps, so we analyzed the expression of ABCB1/MDR1 and ABCG2, two major p-glycoproteins commonly up-regulated in cancer stem cells (CSCs). ABCB1/MDR1 was found to be up-regulated in T47D^OE^ cells but not in MCF-7^OE^ (Figure 2H), whereas ABCG2 was equally expressed in all cell lines (Supplementary Figure 2D). This suggests that although ABCB1/MDR1 may have a role in the chemoresistance of RANK OE cells, other mechanisms will be involved.

Next we questioned if increased expression of mesenchymal, stemness and chemoresistance-related markers is also associated with RANK expression in human ER+ tumors. To address this question, we analyzed the TCGA breast cancer cohort, including all female patients with known ER status (*n* = 1015) (Supplementary Table 2). In this cohort, as expected, *RANK* expression was higher in ER-negative (ER-) tumors vs ER+HER2+/- tumors (Supplementary Figure 2E). Moreover, when considering all cases, RANK^high^ was associated with ER, PR and HER2 negative status (*p* < 0.0001, *p* < 0.0001 and *p* = 0.0273, respectively; dichotomizing patients into RANK^high^ or RANK^low^, according to median *RANK* expression) (Supplementary Table 3). When considering ER+ or ER+HER2- cases, *RANK* expression was not significantly associated with any clinicopathologic characteristics.

We interrogated this cohort for the expression of the genes altered in RANK OE cell lines. RANK^high^ tumors in the ER+HER2- cohort were significantly increased in the expression of *Vimentin*, *Snail*, *Slug*, *TWIST1*, and *β-catenin,* despite differences in *E-cadherin* and *N-cadherin* being non-significant (Figure 2I). We also observed increased expression of *Oct4*, *NANOG* and *ALDH1A3* (Figure 2J), and increased expression of both *ABCB1* and *ABCG2* (Figure 2K). Correlation analysis corroborated these results, with the exception of *NANOG* (Supplementary Table 4). When considering all ER+ cases, independently of HER2 status, we observed the same associations, plus decreased *E-cadherin* in RANK^high^ tumors (Supplementary Figure 2F–2H and Supplementary Table 4).

To further expand our results into the clinical setting, we analyzed a second cohort, in this case a cohort of 57 patients diagnosed with breast cancer at Hospital de Santa Maria (Supplementary Table 5). 33/57 (57.9%) samples were positive for *RANK* expression (RANK+) by RT-qPCR. We analyzed the correlation between the expression of *RANK* and *Vimentin*, *N-cadherin*, *Twist*, *Slug*, *Oct4* and *Cyclin D1* (Supplementary Figure 2I). We observed a significant positive correlation between *RANK* and *Vimentin* (rho = 0.47, *p* = 0.007) and *Slug* (rho = 0.37, *p* = 0.037) (with rho = 0.32, *p* = 0.072 for *N-cadherin*). When considering only ER+RANK+ cases (25/57), we observed a significant positive correlation between *RANK* and *Vimentin* (rho = 0.43, *p* = 0.036) and *N-cadherin* (rho = 0.46, 0.022) (Supplementary Figure 2J).

Although we could not confirm all the associations we observed *in vitro*, which is a limitation of our study, these findings reinforce the hypothesis that RANK is associated with mesenchymal ER+ breast cancers.

### RANK overexpression is associated with decreased proliferation and increased invasiveness *in vivo*

**Figure 3:**
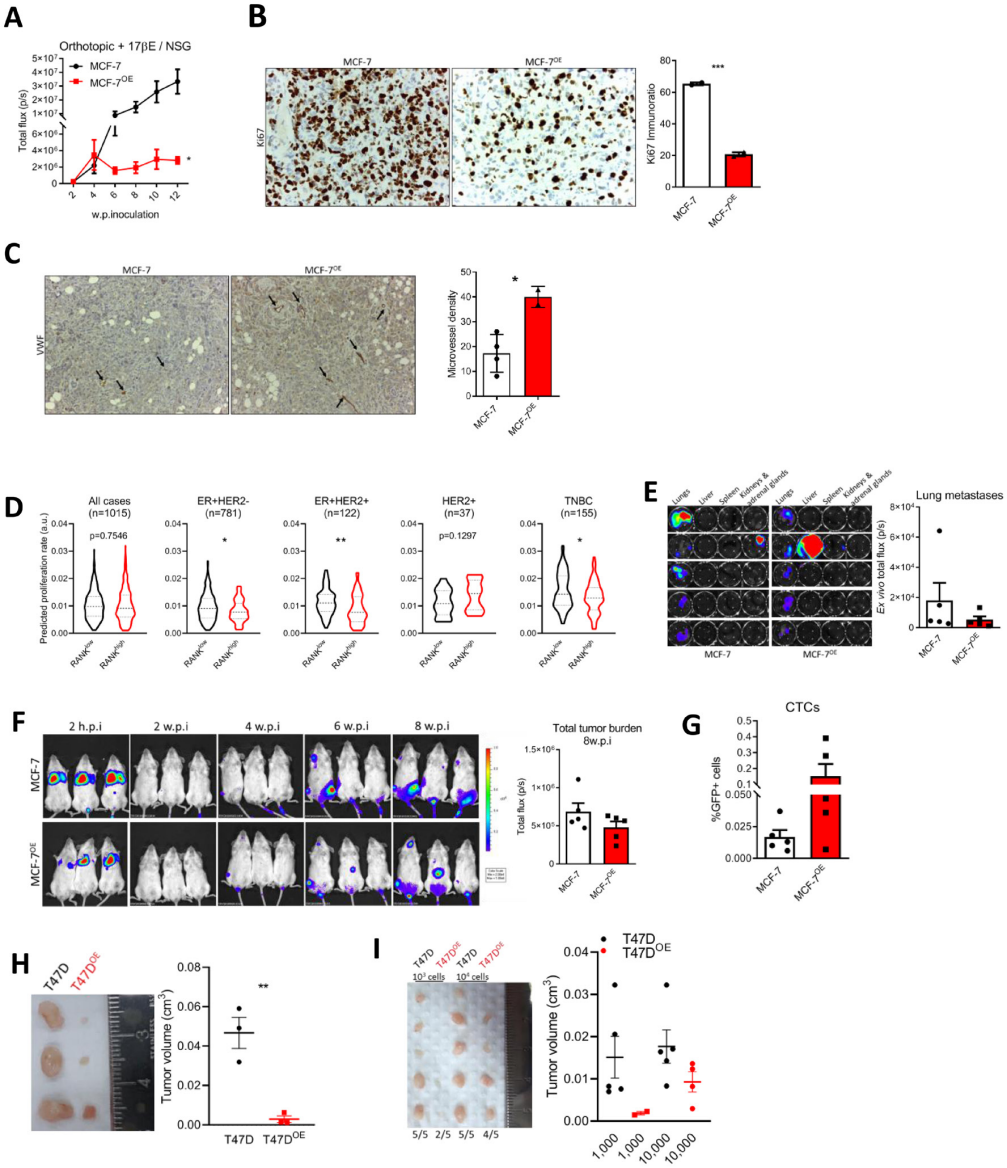
RANK OE cells are less proliferative but more invasive *in vivo*. (**A**) BLI analysis of MCF-7 and MCF-7^OE^ xenografts in NSG mice (*n* = 5–8/group). (**B**) IHC analysis of Ki67. (**C**) Microvessel density (MVD) was quantified after VWF immunohistochemistry. (**D**) Predicted proliferation rate in TCGA cohort according to median *RANK* expression in each sub-group. (**E**) *Ex vivo* BLI of visceral organs and quantification of lung tumor burden. (**F**) BLI analysis of mice inoculated in the tail vein with MCF-7 or MCF-7^OE^ cells (*n* = 5/group). (**G**) Flow cytometry analysis of positive GFP cells in whole blood collected at sacrifice. (**H**, **I**) Tumor volume (Tvol = 1/2 (length × width^2^)) measured at necropsy of T47D or T47D^OE^ adherent cell-derived xenografts (*n* = 3/group) (H) or T47D or T47D^OE^ tumorspheres-derived xenografts (*n* = 5/group) (I). FiJi was used to obtain the best contrast for western blot band visualization, and background was removed for band densitometry analysis. Data is presented as mean ± SEM. ^*^ *p* < 0.05, ^**^ *p* < 0.01, ^***^ *p* < 0.001.

We next aimed to characterize RANK OE cells *in vivo*. First we used an orthotopic xenograft model in NSG mice supplemented with estradiol. MCF-7 RANK OE xenografts were significantly smaller than MCF-7 xenografts (Figure 3A and Supplementary Figure 3A); and had lower Ki67 (Figure 3B), concordant with the slower proliferation rate observed in MCF-7 OE cell line (Figure 1F and Supplementary Figure 1E), but angiongenesis was increased as observed by higher microvessel density (MVD; Figure 3C). RANK expression was confirmed in tumor tissue collected at necropsy (Supplementary Figure 3B), as well as *Twist* and *N-cadherin* up-regulation in RANK OE cells. We also observed that *ESR1* and *PGR* were down-regulated in RANK OE cells growing *in vivo*, which may account for the decreased growth rate and suggests that ER up-regulation *in vitro* may be due to culture-specific conditions, like insulin supplementation. Moreover, RANK OE was accompanied by a slightly higher stromal content (Supplementary Figure 3C).

Difference in xenograft growth was so remarkable that we further confirmed our findings by deriving a second MCF-7 RANK OE cell line (MCF-7^OE2^), this time by CRISPR-Cas9 gene activation (Supplementary Figure 4A). Again, RANK OE2 xenografts were significantly smaller than MCF-7 xenografts (Supplementary Figure 4B); with lower Ki67 (Supplementary Figure 4C) and higher desmoplasia (Supplementary Figure 4D). We confirmed the overexpression of *RANK, N-cadherin*, *Vimentin*, *Snail*, *Slug* and *Twist* in RANK OE2 xenografts (Supplementary Figure 4E). Notably, flow cytometry analysis of xenografts derived from the simultaneous inoculation of RANK OE2 and parental cells (1:1, MIX group) revealed that MIX tumors were enriched in RANK OE2 cells (GFP+RFP+) in the end of the experiment (Supplementary Figure 4F). This may suggest that RANK OE cells have increased fitness in comparison with parental cells, and will be important to determine which factors promote the growth of RANK OE cells in these conditions, and which is the competition mechanism involved in this interaction.

Given these findings, we questioned if human ER+HER2- tumors with elevated RANK expression were also associated with a decreased proliferative index. To address this question we compared the predicted proliferation rate of TCGA breast cancers [24], in RANK^high^ and RANK^low^ tumors, dichotomized according to the median *RANK* expression in each sub-group. RANK^high^ ER+ tumors had significantly lower predicted proliferation rate, when compared to RANK^low^ tumors, independently of HER2 status (Figure 3D). Moreover, predicted proliferation rate was also lower in TNBC RANK^high^ tumors. When considering all tumors or HER2+ tumors, differences were not significant.

Next, to assess *in vivo* the metastatic potential of RANK OE cells we used a metastasis experimental model via tail vein inoculation of cancer cells in NSG mice supplemented with exogenous estradiol. Lung metastases-specific burden (Figure 3E) and total tumor burden (Figure 3F) were identical between MCF-7 and RANK OE inoculated mice. Moreover, liver and spleen metastases were observed in one animal only, in RANK OE group (Supplementary Figure 5A, 5B). However, because orthotopic MCF-7 and MCF-7^OE^ cells did not metastasized spontaneously, we cannot compare proliferation rate in primary and metastatic tumors. Importantly, mice inoculated with RANK OE cells had more CTCs (Figure 3G).

Finally, to confirm that these results were not cell line specific, we analyzed the growth of T47D RANK OE cells *in vivo*. T47D parental or RANK OE cells were inoculated orthotopically and bilaterally in NSG mice supplemented with exogenous estradiol, and tumor volume was measured *ex vivo* eight weeks post-inoculation. As previous, RANK OE xenografts were significantly smaller than parental counterparts (Figure 3H and Supplementary Figure 6A). However, 10.000 tumorsphere-derived cells were able to originate bigger RANK OE xenografts than inoculation of 2 × 10^6^ adherent cells, which was not observed for the parental counterparts (Figure 3I and Supplementary Figure 6C). This suggests that selection of specific RANK OE clones by non-adherent culture conditions may trigger faster tumor growth. In all models, no differences in mice body weight between groups were observed (Supplementary Figures 3D, 4G, 5C, 6B, 6D).

### Continuous RANKL exposure has a negative feedback effect on RANK pathway and induces HR loss in ER+HER2- cells

**Figure 4:**
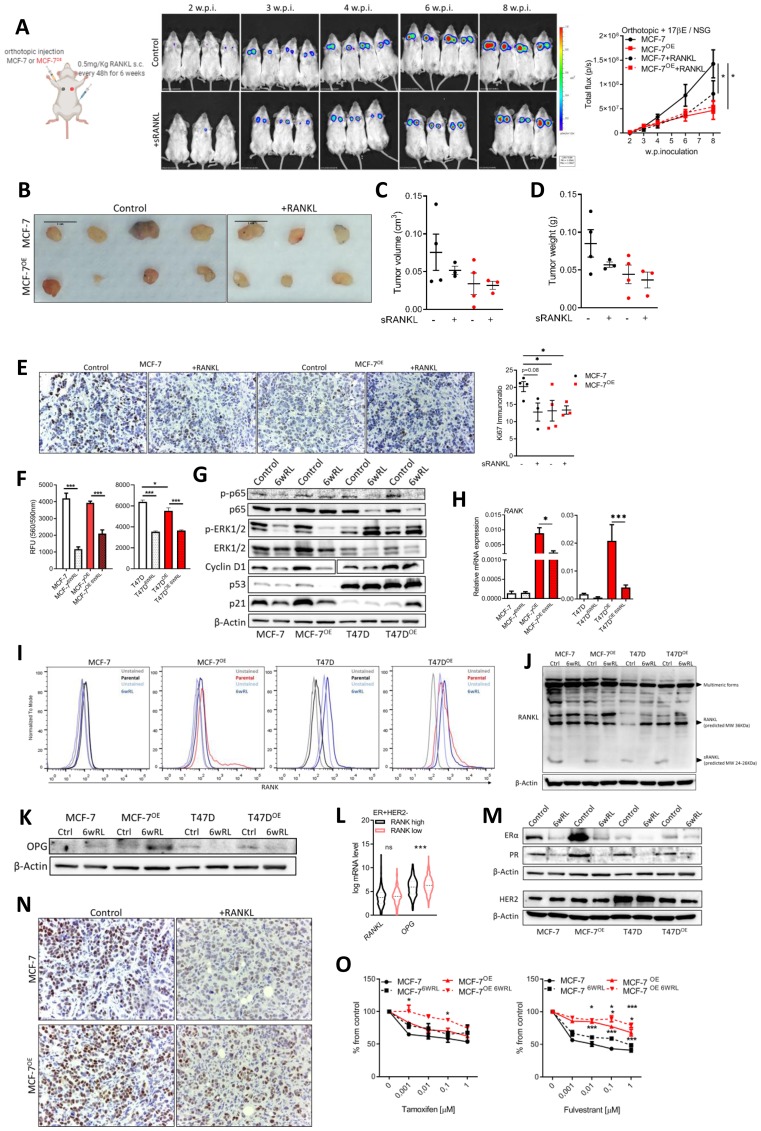
Continuous RANKL decreases cell proliferation and ER expression. (**A**) BLI analysis of MCF-7 and MCF-7^OE^ xenografts in NSG mice (*n* = 3–4/group). (**B**) Tumors photographed at necropsy. (**C**) Tumor volume (Tvol = 1/2 (length × width^2^)) measured at necropsy. (**D**) Tumor weight at necropsy. (**E**) IHC analysis of Ki67. (**F**) Cell viability was measured 6 weeks after exposure to RANKL (*n* = 3). (**G**) Western blot of RANK downstream targets and cell cycle control proteins with β-Actin as loading control. (**H**) RT-qPCR of *RANK* (*n* = 3). (**I**) Flow cytometry of RANK. (**J**, **K**) Western blot of indicated proteins with β-Actin as loading control. (**L**) Interest gene expression in the ER+HER2- TCGA cohort (*n* = 587) according to RANK expression. (**M**) Western blot of indicated proteins with β-Actin as loading control. (**N**) Representative images of IHC of ER. (**O**) Cell viability was measured 7 days after exposure to the indicated drugs, with medium replacement every 48 h. (*n* = 3). FiJi was used to obtain the best contrast for western blot band visualization, and background was removed for band densitometry anslysis. Data is presented as mean ± SEM. ^*^ *p* < 0.05, ^**^ *p* < 0.01, ^***^ *p* < 0.001.

Since RANKL was not up-regulated in RANK OE cells, and despite RANKL not affecting the proliferation of cells *in vitro*, we interrogated if low environmental RANKL could be limiting the proliferation of RANK OE cells. To test this hypothesis we used an orthotopic xenograft model in NSG mice supplemented with exogenous estradiol plus RANKL supplementation by sub-cutaneous injection every 48 h. Surprisingly, not only supplementation with RANKL did not increase the growth of RANK OE xenografts, but instead it decreased the growth of parental MCF-7 xenografts (Figure 4A–4D), which had lower Ki67 in mice supplemented with RANKL (Figure 4E). RANKL supplementation did not affected mice body weight (Supplementary Figure 7A), and although using a low-dosage 0.5 mg/KgBW sRANKL every 48 h, to answer if exogenous RANKL affected the bone physiology, we quantified TRAcP 5b in the serum of mice, and observed no differences between groups (Supplementary Figure 7B).

To investigate why RANKL decreased the growth of MCF-7 xenografts, we cultured MCF-7, T47D, and their RANK OE counterparts *in vitro*, in the presence of RANKL for six weeks. At the end of this period we measured cell proliferation, which was lower in cells continuously exposed to RANKL (Figure 4F). Moreover, p-p65, p-ERK and Cyclin D1, downstream of RANK, were decreased suggesting RANK pathway inhibition (Figure 4G). p-ERK was increased in T47D cells only, suggestive of a compensatory mechanism, maybe related with oncogenic p53. p53 and p21 were also down-regulated in MCF-7 cells exposed to RANKL (Figure 4G). We hypothesized that continous RANKL exposure was exerting a negative feedback effect, and to address this hypothesis we measured RANK expression, which was found to be down-regulated in RANKL-exposed cells (Figure 4H, 4I). Additionally, although RANKL levels were identical in all cell lines, the expression of the secreted form of RANKL was not observed in cells exposed to continuous RANKL (Figure 4J). In accordance with a negative effect over autocrine RANK activation, OPG, which is a soluble decoy of RANKL, was up-regulated in MCF-7 RANKL-exposed cells (Figure 4K) and in ER+HER2- RANK^high^ human tumors (Figure 4L).

Since *in vivo* growth of ER+ cells is highly dependent on estradiol and ER signaling, we also questioned if continuous RANKL affected ER and/or PR. Although up-regulated in RANK OE cells, ER and PR were highly down-regulated in all cells exposed to RANKL (Figure 4M), as well as in xenografts growing under RANKL supplementation (Figure 4N). Moreover, ER loss resulted in decreased sensitivity to fulvestrant (Figure 4O), even in fulvestrant-resistant RANK OE cell lines. Furthermore, we confimed that RANK OE cells were also dependent on ER signaling *in vivo*, originating even smaller xenografts in the absence of exogenous estradiol supplementation (Supplementary Figure 7C, 7D, 7F), which induced a strong ER downregulation (Supplementary Figure 7E).

## DISCUSSION

The pharmacological inhibition of RANK pathway has gained particular attention in adjuvant control of bone recurrence from breast cancer or in the prevention of BRCA-related breast cancer, since its role in bone remodeling and breast carcinogenesis was clearly shown.

Assessment of RANK expression in clinical samples has been sparse, but consistently associated with HR-negative status [13, 17, 19]. However, it has been reported the existence of RANK-positive ER+ breast cancers, like in GeparTrio trial cohort (*n* = 601) where 23 out of 160 RANK-positive cases were ER+ [19]. Despite RANK signaling has been implicated in cell dedifferentiation and cancer progression, depending on cellular type [13, 21], the significance of RANK OE in ER+ breast cancer cells was never assessed.

In this study we demonstrate that as previously described for TNBC, RANK OE in ER+HER2- cell lines induced the acquisition of mesenchymal traits, suggesting a more invasive behavior; and the onset of a stem cell-like population. Moreover, fulvestrant, which is part of the current standard of care therapy for ER+HER2- breast cancer, was less effective in these cells, which may accumulate as therapy-resistant clones and contribute to cancer progression. *In vivo*, RANK OE cells had a severely decreased proliferation rate but were more invasive. Importantly, these cells, and even ER+HER2- cells with low RANK expression, were particularly sensitive to a RANKL rich environment, which induced ER downregulation and exacerbated resistance to HT. By analyzing the TCGA breast cancer cohort and an independent clinical cohort of breast cancer tumors, we could validate our findings in human samples, as we observed an association between *RANK* expression and mesenchymal, stemness and chemoresistance-related genes; as well as an association between higher *RANK* and decreased predicted proliferation rate. These findings pave the way to study the effectiveness of RANK pathway inhibition, as a way to improve ER+HER2- breast cancer outcomes.

The expression of stem cell markers upon induction of EMT has been described in different models and enables self-renewal of cancer cells that disseminate from a primary tumor. RANK OE cells had increased ability to form spheres in non-adherent conditions, a property associated with MaSCs [25] and CSCs [26, 27]. These characteristics are in accordance with the association between RANK expression and mesenchymal- and stem cell-related genes we found in human breast tumors.

Consistent with a CSC-like phenotype, RANK OE cells were chemoresistant. However, only T47D^OE^ cells had an increase in MDR1 (P-gp/ABCB1/MDR1), associated with chemoresistance to taxanes and anthracyclines, and poor outcomes in breast cancer [28, 29]. The closely related ABC transporter ABCG2/BCRP was not overexpressed in any of the RANK OE cells in our study. Therefore, other mechanisms, like the decreased proliferation rate, EMT [25], or the expression of negative regulators of apoptosis [30], may be implicated; although MDR1 and ABCG2 were up-regulated in RANK^high^ ER+HER2- human breast tumors.

An important potential therapeutic implication of our study is the finding that RANK OE cells are less sensitive to fulvestrant. Despite the efficacy of endocrine therapy in ER+ breast cancer, a significant proportion of patient’s present intrinsic or acquired resistance, leading to poor outcomes. Different mechanisms of resistance to fulvestrant have been proposed, including ER mutation or loss, activation of PI3K pathway, aberrant expression of cell cycle regulatory proteins, EGFR and HER2 amplification, and activation of ErbB, IGF or NF-kB pathways [31]. We hypothesize that the presence of ER+ RANK-positive cells within a tumor, with higher ER expression and sustained NF-kB pathway activity, may represent an important reservoir of HT-resistant cells, which may have the skills to survive therapies that kill HT-sensitive tumor cells.

Lower proliferation rate of RANK OE cells was remarkable *in vivo* in orthotopic models, despite estradiol supplementation. Importantly, we also found that elevated RANK expression was associated with decreased predicted proliferation rate, in human ER+HER2+/- and TNBC breast tumors from the TCGA database. This is an important observation, since a previous analysis of a cohort of 66 breast adenocarcinomas has found RANK mRNA to be higher in tumors with high Ki67 (> 40%) [13]. This reinforces the idea that the way RANK expression affects cell proliferation may depend on breast cancer sub-type and specific environmental characteristics.

RANK OE xenografts were also characterized by high desmoplasia, which has been associated with disease progression and poor outcome in different solid tumors, including breast cancer, as partly determinative of metastatic capacity [32–34].

ER+ RANK-positive cells will most likely coexist with other cell types in a heterogeneous tumor. In mixed xenografts RANK OE cells were the most abundant at sacrifice. Therefore, RANK OE clones with increased tumorigenic ability may be selected by environmental pressure. In fact, we show that 10,000 tumorsphere-derived T47D RANK OE cells were able to form bigger xenografts than 2 × 10^6^ adherent cells. This suggests the selection of a more proliferative ‘unlocked’ population by anchorage-independent growth. It will be important to unravel what may trigger the growth of RANK-positive ER+ cells, as well as if inhibition of RANKL-RANK pathway may eliminate or control these cells.

Although we hypothesize that the microenvironment is particularly relevant for the proliferation of ER+ RANK-positive cells, RANKL was not the limiting factor sustaining low proliferation *in vivo*. Parental and RANK OE cells express identical levels of endogenous RANKL, and short-term exposure to exogenous RANKL did not affect proliferation, as previously reported [20, 22]. However and surprisingly, continuous exposure to RANKL, not only did not rescued the growth of RANK OE cells, but it was able to decrease the proliferation and ER expression in parental MCF-7 cells *in vivo*. We found that long-term and continuous exposure to RANKL activated an autocrine negative feedback mechanism, leading to down-regulation of RANK and RANK pathway, and up-regulation of OPG. OPG is a soluble decoy of RANKL that we also show to be significantly increased in patients with RANK^high^ ER+HER2- breast cancers. It has been reported that high OPG mRNA correlates with better prognosis in breast cancer patients [17]. However, OPG expression in breast cancer cells, including MCF-7 and T47D, has been shown to be associated with inhibition of TRAIL-induced apoptosis *in vitro*; increased proliferation via binding to various cell surface receptors; induction of angiogenesis; and paracrine regulation of non-tumoral cells in the tumor microenvironment [35]. Therefore, OPG up-regulation deserves further studies to clearly assess its contribution to breast cancer progression.

We also observed that the secreted form of RANKL was not present in cells exposed to RANKL. It has been shown that the soluble form of RANKL is not necessary for bone remodeling [36]; but it promotes the formation of tumor metastases in bone [37]. Overall, these data suggest that a negative feedback mechanism attenuates continuous RANK activation in ER+HER2- breast cancer cells. However, this also induced ER and PR down-regulation, and increased resistance to HT, potentially providing a second driver of cellular quiescence and resistance to therapy. Therefore, we hypothesize that a RANKL rich environment, like in post-menopausal women [38] or the bone microenvironment [6], may contribute for ER loss and resistance to HT in ER+ tumors, independently of elevated RANK expression.

In conclusion, our results demonstrate that ER+ RANK-positive cells may drive resistance to chemotherapy and HT and contribute to metastization; and pave the way to study the effectiveness of RANK pathway inhibition, as a way to improve ER+HER2- breast cancer outcomes.

## MATERIALS AND METHODS

### *In silico* analysis

Normalized gene expression [log2 FPKM (fragments per kilobase million)] and clinical data for 1015 breast tumors from The Cancer Genome Atlas (TCGA) breast cancer dataset (https://portal.gdc.cancer.gov/) were retrieved from UCSC XENA (https://xena.ucsc.edu/). Demographic and clinicopathological characteristics were tabulated according to the full cohorts and to *RANK* status (high or low, dichotomized according to *RANK* median expression in each cohort). Normalized *RANK* (*TNFRSF11A*) expression [log2 RPKM (reads per kilobase million)] in breast cancer cell lines was derived from the Cancer Cell Line Encyclopedia (CCLE) database (https://portals.broadinstitute.org/ccle). Analyses were performed using GraphPad Prism8 software.

### Human samples

In this retrospective cohort study we included 57 patients diagnosed with breast cancer and treated at the Medical Oncology Department of Hospital de Santa Maria, for which frozen tumor sections were available at Biobanco-iMM. Clinical information was retrospectively collected. Ethical approval for this study was granted by institutional review boards.

### Cell culture

Human breast carcinoma cell lines MCF-7^GFP+Luc+^ and MDA-MB-231^GFP+Luc+^ (herein designated by MCF-7 and MDA-MB-231, respectively) were provided by Dr. Sérgio Dias (Instituto de Medicina Molecular, Lisbon, Portugal); and T47D cell line was provided by Dr. Phyllippe Clézardin (INSERM, Lyon, France). Cells were cultured under standard conditions, used at low passage number, and tested for *Mycoplasma* contamination. For continuous RANKL exposure experiments, medium was supplemented with 1 μg/ml RANKL (#11000457, Amgen Inc.) every 48 h for 6 weeks. Doubling time was calculated in GraphPad Prism 8 software, using the exponential growth equation with least squares regression fitting model.

### RANK (TNFRSF11A) overexpression

For RANK overexpression cells were transduced with RANK lentiviral overexpression particles (RANK (*TNFRSF11A*) overexpression plasmid pReceiver-Lv121 (#EX-O0007-Lv121, GeneCopoeia)), and selected with 0.5 μg/mL (MCF-7 and MDA-MB-231) or 1.5 μg/ml (T47D) puromycin dihydrochloride (#sc-108071, Sigma-Aldrich). For CRISPR/Cas9 activation of RANK expression (MCF-7^OE2^), MCF-7 cells were transduced with huRANK lentiviral activation particles (#sc-400559-LAC, Santa Cruz Biotechnology) or control lentiviral particles (#sc-437282, Santa Cruz Biotechnology), and selected with 0.5 μg/mL puromycin dihydrochloride, 5 μg/ml blasticidin S HCl (#sc-495389, Santa Cruz Biotechnology) and 200 μg/mL hygromycin B (#sc-29067, Santa Cruz Biotechnology). RANK overexpression was confirmed by RT-qPCR and flow cytometry. Next MCF-7^OE2^ cells were transduced with Cignal Lenti Positive Control (RFP) ready-to-transduce lentiviral particles (#336891, Quiagen), and selected with 0.5 μg/mL puromycin dihydrochloride, followed by RFP+ cell sorting in a FACS Aria III cell sorter (BD Biosciences).

### RT-qPCR

Cells or mouse tumors total RNA was extracted using the NZY Total RNA Isolation kit (#MB13402, Nzytech). RNA from human breast tumors was obtained from Biobanco-iMM. DNase I-treated RNA was reverse transcribed using the NZY M-MuLV First-Strand cDNA Synthesis kit (#MB17301, Nzytech) and Oligo (dT)20 primer; and cDNAs were amplified by real-time PCR using TaqMan Gene Expression Master Mix (#4369016, Applied Biosystems) or NZY qPCR Green, ROX (#MB22003, Nzytech). Specific primers included: *TNFRSF11A* (#Hs00921372_m1, Applied Biosystems), *TNFSF11* (#Hs00243522_m1, Applied Biosystems), *PGR* (#Hs01556702_m1, Applied Biosystems), *ESR1* (#Hs01046816_m1, Applied Biosystems), *Twist* (Fw: 5′-ccggagacctagatgtcattg-3′; Rv: 5′-ccacgccctgtttctttg-3′), *Slug* (Fw: 5′-ccaaactacagcgaactgga-3′; Rv: 5′-gtggtatgacaggcatggag-3′), *Vimentin* (Fw: 5′-gaaaacaccctgcaatctt-3′; Rv: 5′-cctggatttcctcttcgtg-3′), *N-cadherin* (Fw: 5′-tatcgaaggatgtgcatga-3′; Rv: 5′-caggctcactgctctcata-3′), *Oct4* (Fw: 5′-ctgagggcgaagcaggagtc-3′; Rv: 5′-cttggcaaattgctcgagtt-3′) and *GAPDH* (#PPH00150F, SA Biosciences). Gene expression was normalized using the housekeeping gene *GAPDH*, and relative mRNA expression was calculated using the 2^–ΔΔCt^ method.

### Western blotting

For analysis of RANK pathway activation by RANKL, cells were serum-starved in low-serum medium (0.1% FBS) for 24 h, and stimulated with 1 μg/mL human RANKL (#11000457, Amgen Inc.) for the indicated time points. Specific primary antibodies included: mouse monoclonal anti β-Actin antibody (#ab6276; Abcam), rabbit monoclonal anti α-Tubulin (11H10) (#2125, Cell Signaling), rabbit polyclonal anti-NFkB p65 (#ab16502, Abcam), rabbit monoclonal anti-NF-kB p65 (D14E12) (#8242, Cell Signaling), rabbit monoclonal anti-Phospho-NF-kB p65 (Ser536) (93H1) (#3033, Cell Signaling), mouse monoclonal anti-IkBα (L35A5) (#4814, Cell Signaling), rabbit monoclonal anti-Phospho-IkBα (Ser32) (14D4) (#2859, Cell Signaling), rabbit polyclonal anti-Phospho-ERK1/2 (Thr-202/Tyr-204) (#sc-1682, Santa Cruz Biotechnology), rabbit polyclonal anti-ERK1/2 (c-14) (#sc-154, Santa Cruz Biotechnology), rabbit polyclonal anti-Phospho-AKT1/2/3 (Ser-473) (D9E) (#sc-7985, Santa Cruz Biotechnology), rabbit polyclonal anti-AKT1/2/3 (H-136) (#sc-8312, Santa Cruz Biotechnology), rabbit monoclonal anti-Vimentin (D21H3) (#5471, Cell Signaling), rabbit monoclonal anti-N-Cadherin (D4R1H) (#13116, Cell Signaling), rabbit monoclonal anti-β-Catenin (D10A8) (#8480, Cell Signaling), rabbit monoclonal anti-Snail (C15D3) (#3879, Cell Signaling), rabbit monoclonal anti-Slug (C19G7) (#9585, Cell Signaling), rabbit monoclonal anti-E-Cadherin (24E10) (#3195, Cell Signaling), mouse monoclonal anti-Nanog (hNanog.2) (#145768-80, eBioscience), mouse monoclonal anti-Sox2 (245610) (#MAB2018, R&D systems), mouse monoclonal anti-OCT4 (7F9.2) (#MAB4419, Millipore), rabbit monoclonal anti-Phospho-Rb (Ser807/811) (D20B12) (#8516, Cell Signaling), rabbit monoclonal anti-p21 Waf1/Cip1 (12D1) (#29475, Cell Signaling), rabbit monoclonal anti-Cyclin D1 (92G2) (#2978, Cell Signaling), mouse monoclonal anti-Cyclin E (HE12) (#05-363, Sigma-Aldrich), rabbit monoclonal anti-CDK2 (78B2) (#2546, Cell Signaling), rabbit monoclonal anti-CDK4 (D9G3E) (#12790, Cell Signaling), mouse monoclonal anti-CDK6 (DCS83) (#3136, Cell Signaling), rabbit monoclonal anti-p27 Kip1 (D69C12) (#3686T, Cell Signaling), mouse monoclonal anti-p53 (Bp53-12) (#sc-263, Santa Cruz Biotechnology), mouse monoclonal anti-p18 INK4C (DCS118) (#2896, Cell Signaling), rabbit monoclonal anti-ERα (D6R2W) (#132585, Cell Signaling), mouse monoclonal anti-PR (#KMC912, eBioscience), rabbit monoclonal anti-Her2/ErbB2 (#22425, Cell Signaling), rabbit monoclonal anti-BCRP/ABCG2 (#ab108312, Abcam), rabbit polyclonal anti-hsRANKL (#500-P133, PeproTech), goat polyclonal anti-OPG (#AF805, R&D Systems). Horseradish peroxidase (HRP)-conjugated specific secondary antibodies anti-mouse-HRP IgG (#012018, Cell Signaling), anti-rabbit-HRP IgG (#022019, Cell Signaling), and anti-goat-HRP IgG (#sc-2354, Santa Cruz Biotechnology) were used. Band intensity was calculated using ImageJ software and normalized for β-Actin.

### Flow cytometry

For RANK expression analysis, trypsinized cells were incubated with mouse monoclonal antibody anti-RANK (#M331, Amgen Inc.) and labelled with 1:100 Cy5 conjugated AffiniPure goat anti-mouse IgG (#115-175-205, Dianova). For ABACB1/MDR1 expression analysis, trypsinized cells were incubated with biotinylated mouse antibody anti-CD243 (#348602, Milteny Biotec) and labelled with anti-biotin-PE antibody (#130-111-068, Miltenyi Biotech). For cell cycle analysis cells were cultured under standard conditions for 24 h (asynchronous), synchronized by 24 h serum starvation, and released from serum starvation for another 24 h. Cell cycle analysis was performed using the Propidium iodide (PI) flow cytometry kit (#ab139418, Abcam). Analysis was made using FlowJo V10 software.

### ELISA assays

sRANKL in conditioned media was quantified by sandwich ELISA, using 1 μg/ml polyclonal rabbit anti-sRANKL antibody (#500-P133, PreproTech) as capture antibody, 0.5 μg/ml biotinylated rabbit anti-sRANKL antibody (#500-P133BT, PreproTech) as detection antibody, and ABTS (2,2′-Azinobis [3-ethylbenzothiazoline-6-sulfonic acid]-diammonium salt) as substrate (#900-K00, PreproTech). RANKL (#11000457, Amgen Inc.) was used as standard; and protein concentration was normalized for media concentration factor and number of cells.

TRAcP 5b was quantified in mouse serum using the MouseTRAP (TRAcP 5b) ELISA kit (#SB-TR103, IDS), according to the manufacturer instructions.

### Tumorsphere formation assay

Cells were seeded in 3D Tumorsphere Medium XF (#C-39670, PromoCell) in ultra-low attachment 6-well plates. After 7 days, average tumorsphere area was calculated by measuring all tumorspheres > 50 μm in diameter per well. Sphere Forming Capacity (SFC) (%) was determined as the number of mammospheres > 50 μm/number of cells seeded) × 100. 3D tumor spheroid invasion assay was performed as previously described [39], using 2.5 μg/ml RANKL (#11000457, Amgen Inc.).

### Viability assays

Cells were seeded in 96 well-plates, with or without paclitaxel (#Y0000698, Sigma-Aldrich), doxorubicin hydrochloride (#D2975000, Sigma-Aldrich), tamoxifen (#HT904, Sigma-Aldrich), or fulvestrant (#S1191, Selleckchem). Medium was replaced every two days. After 72 h for paclitaxel and doxorubicin, and seven days for tamoxifen and fulvestrant, to assess the proliferative effect of estrogen, cells were cultured in 24-well plates, in phenol red-free DMEM:F12 medium (# 11039021, Gibco) supplemented with 5% (v/v) charcoal stripped FBS (csFBS, #12676029, Gibco), for five days, with or without 10 nM β-estradiol (#E2758, Sigma-Aldrich). Viability was assessed by Alamar blue assay (#DAL1100, Invitrogen).

### Migration assay

Migration of cancer cells was assessed using a 96-well chemotaxis chamber with polycarbonate filters (8 mm pore size) (#106-8, Neuro Probe), as previously described [22]. Briefly, cells were serum-starved for 24 h and stimulated with 2.5 μg/ml RANKL, neutralized or not with 2.5 mg/ml anti-hTRANCE/TNFSF11 antibody (#MAB626, R&D Systems), for 24 h. Cells were fixed with 2%PFA, stained with crystal violet and images acquired using a Leica DM750 bright field microscope, with 40× magnification. Cells were counted using ImageJ.

### Mouse models

All animal experiments were reviewed and approved by the Institutional Animal Welfare Body of the Institute of Molecular Medicine, and licensed by the national regulatory agency Direcção Geral de Alimentação e Veterinária (DGAV). Experiments were performed in four week old NOD scid gamma (NSG) mice (Charles River) supplemented with subcutaneous 17β-estradiol pellets (#SE-121, Innovative Research of America), unless otherwise stated. For orthotopic xenografts, cells were resuspended in 50% phenol-free matrigel solution (#7338015, Corning). For the experimental metastases model NSG mice were inoculated in the tail vein with 2.5 × 10^6^ cells/ml. Tumor growth was monitored weekly by luminescence analysis. For circulating tumor cells (CTC) analysis, venous blood was collected by cardiac puncture before sacrifice, and analyzed for GFP and RFP expression in a BD LSRFortessa flow cytometer (BD Biosciences).

### Immunohistochemistry

5 μm tissue sections from Formalin-Fixed Paraffin-Embedded (FFPE) samples were stained by immunohistochemistry (IHC) for the detection of Ki67, ER and VWF. Specific antibodies included rabbit anti-human Ki67 primary antibody (1:100, MIB-1, Dako), rabbit anti-human ERα (RTU, EP-1, Dako) and polyclonal rabbit anti-human von Willebrand Factor (VWF) primary antibody (1:200, #A0082, Dako), and EnVisionTM Detection System, rabbit/mouse (#411083, Dako). Ki67 was quantified as the percentage of DAB-stained nuclear area over total nuclear area (hematoxylin-stained nuclei regions), from 5 fields at 400× magnification, using the ImageJ software. Microvessel density (MVD) was determined using VWF IHC. A single microvessel was defined as previously described [40], and microvessels were quantified in 4 fields at 200× magnification.

### Statistical analysis

Data was analyzed using GraphPad Prism8 software. The unpaired *t*-test, one-way or two-way ANOVA were used as appropriate. Results are presented as mean ± SEM and *p*-value < 0.05 was considered statistically significant.

## SUPPLEMENTARY MATERIALS





## Abbreviations

3D: three dimensions
CCLE: Cancer Cell Line Encyclopedia
cDNA: complementary DNA
CTC: circulating tumor cells
DAB: 3,3′-Diaminobenzidine
DFS: disease-free survival
DGAV: Direcção Geral de Alimentação e Veterinária
ELISA: enzyme-linked immunosorbent assay
EMT: epithelial-mesenchymal transition
ER: estrogen receptor
ER+: ER-positive
FBS: fetal bovine serum
csFBS: charcoal stripped FBS
FFPE: Formalin-Fixed Paraffin-Embedded
FPKM: Fragments Per Kilobase Million
GFP: green fluorescent protein
HR: hormone receptor
HRP: horseradish peroxidase
HT: hormone therapy
IHC: immunohistochemistry
iMM: Instituto de Medicina Molecular
LUC: luciferase
MaSCs: mammary stem cells
MCF-7: Michigan Cancer Foundation-7
MECs: mammary epithelial cells
mRNA: messenger RNA
MVD: microvessel density
NSG: NOD scid gamma mice
PE: phycoerythrin
PFA: paraformaldehyde
PI: propidium iodide
PR: progesterone receptor
qPCR: quantitative polimerase chain reaction
RANK OE: RANK overexpression
RANKL: receptor activator of nuclear factor-kB ligand
RFP: red fluorescent protein
RNA: ribonucleic acid
RPKM: Reads Per Kilobase Million
RTU: ready-to-use
SEM: standard error of the mean
SFC: Sphere Forming Capacity
TCGA: The Cancer Genome Atlas
TNBC: triple negative breast cancer
TNFR: tumor necrosis factor receptor
VWF: von Willebrand Factor
