# Supplementary material for: Expression of receptor activator of NFkB (RANK) drives stemness and resistance to therapy in ER+HER2- breast cancer
Source: Oncotarget. 2020 May 12;11(19):1714–28. doi: 10.18632/oncotarget.27576 (PMC7233807; doi:10.18632/oncotarget.27576)
Supplement: Supplementary file 1 [file oncotarget-11-1714-s001.pdf]

Expression of receptor activator of NFkB (RANK) drives stemness and resistance to therapy in ER+HER2- breast cancer

SUPPLEMENTARY MATERIALS

Cell culture

Human breast carcinoma cell lines MCF-7$^{GFP+Luc+}$ and MDA-MB-231$^{GFP+Luc+}$ (herein designated by MCF-7 and MDA-MB-231, respectively) were provided by Dr. Sérgio Dias (Instituto de Medicina Molecular, Lisbon, Portugal); and T47D cell line was provided by Dr. Phyllippe Clézardin (INSERM, Lyon, France). Cells were cultured in Dulbecco's Modified Eagle's Medium (DMEM, # 41966-029, Gibco) supplemented with 10% (v/v) fetal bovine serum (FBS, #10270106, Gibco), 0,01 mg/mL insulin (#12585014, Gibco), and 1% (v/v) Penicillin/Streptomycin (10,000 U/mL Penicillin, 10,000 µg/mL Streptomycin, # 15140122, Gibco). Cells were maintained at 37°C with 5% CO2, used at low passage number, and tested for *Mycoplasma* contamination by qPCR. For continuous RANKL exposure experiments, medium was supplemented with 1 µg/ml RANKL (#11000457, Amgen Inc.) every 48 h for 6 weeks. For doubling time determination, 1.5×10^4 cells/mL were cultured in T25 flasks under standard conditions, and counted using a hemocytometer 12 h, 24 h, 28 h, 32 h and 36 h after seeding. Doubling time was calculated in GraphPad Prism 8 software, using exponential growth equation with least squares regression fitting model.

RANK (TNFRSF11A) overexpression

For lentiviral transduction, MCF-7, T47D and MDA-MB-231 cells were seeded in 6-well-plates, at a density of 2×10^5 cells/well. 24 hours after seeding, medium was replaced by fresh medium containing RANK lentiviral overexpression particles (RANK (*TNFRSF11A*) overexpression plasmid pReceiver-Lv121 (#EX-O0007-Lv121, GeneCopoeia)). Cells were selected with 0.5 µg/mL (MCF-7 and MDA-MB-231) or 1.5 µg/ml (T47D) puromycin dihydrochloride (#sc-108071, Sigma-Aldrich), starting three days after transduction. For CRISPR/Cas9 activation of RANK expression, MCF-7 cells were seeded in 24-well plates, at a density of 4×10^4 cells/well. 24 hours after seeding, medium was replaced by fresh medium containing 8 µg/ml Polybrene (#sc-134220, Sigma-Aldrich) and huRANK lentiviral activation particles (#sc-400559-LAC, Santa Cruz Biotechnology) or control lentiviral particles (#sc-437282, Santa Cruz Biotechnology). Cells were selected with 0,5 µg/mL puromycin dihydrochloride (#sc-108071, Sigma-Aldrich), 5 µg/ml blasticidin S HCl (#sc-495389, Santa Cruz Biotechnology) and 200 µg/mL hygromycin B (#sc-29067, Santa Cruz Biotechnology), starting three days after transduction. RANK overexpression was confirmed by RT-qPCR and flow cytometry. Next MCF-7^{OE2} cells (RANK OE GFP+Luc+) were transduced with Cignal Lenti Positive Control (RFP) ready-to-transduce lentiviral particles (#336891, Quiagen), selected with 0.5 µg/mL puromycin dihydrochloride (#sc-108071, Sigma-Aldrich), followed by RFP+ cell sorting in a FACS Aria III cell sorter (BD Biosciences).

RT-qPCR

For RT-qPCR analysis of mRNA expression, cells were grown up to 80–90% confluency and total RNA was extracted using the NZY Total RNA Isolation kit (#MB13402, Nzytech), according to manufacturer's instructions. Total RNA from human breast tumors was obtained from Biobanco-iMM. Total RNA was quantified in a NanoDrop spectrophotometer (Thermo Ficsher Scientific), and treated with RQ1 RNase-free DNase I (#M6101, Promega) for 30 min at 37°C, according to manufacturer's instructions. DNase I-treated RNA was reverse transcribed using the NZY M-MuLV First-Strand cDNA Synthesis kit (#MB17301, Nzytech) and Oligo(dT)20 primer, following the manufacturer's protocol. cDNAs were amplified by real-time PCR using TaqMan Gene Expression Master Mix (#4369016, Applied Biosystems) or NZY qPCR Green, ROX (#MB22003, Nzytech), according to manufacturer's instructions. Specific primers included: *TNFRSF11A* (#Hs00921372_m1, Applied Biosystems), *TNFSF11* (#Hs00243522_m1, Applied Biosystems), *PGR* (#Hs01556702_m1, Applied Biosystems), *ESR1* (#Hs01046816_m1, Applied Biosystems), *Twist* (Fw: 5′-ccggagacctagatgtcattg-3′; Rv: 5′-ccacgccctgtttctttg-3′), *Slug* (Fw: 5′-ccaaactacagcgaactgga-3′; Rv: 5′-gtggtatgacaggcatggag-3′), *Vimentin* (Fw: 5′-gaaaacaccctgcaatctt-3′; Rv: 5′-cctggatttcctcttcgtg-3′), *N-cadherin* (Fw: 5′-tatcgaaggatgtgcatga-3′; Rv: 5′-caggctcactgctctcata-3′), *Oct4* (Fw: 5′-ctgagggcgaagcaggagtc-3′; Rv: 5′-cttggcaaattgctcgagtt-3′) and *GAPDH* (#PPH00150F, SA Biosciences). Gene expression was normalized using the housekeeping gene *GAPDH*, and relative mRNA expression was calculated using the $2^{-\Delta\Delta Ct}$ method.

Western blotting

For analysis of RANK pathway activation by RANKL, 4×10^5 cells were seeded in 6-well plates for 24 h, serum-starved in low-serum medium (0,1% FBS, 1% Pen/Step) for 24 h, and stimulated with 1 μg/mL human RANKL (#11000457, Amgen Inc.) for the indicated time points. Total cell lysates were prepared with RIPA buffer (#R0278, Sigma-Aldrich) containing 1:100 protease (#5871, Cell Signaling) and phosphatase (#P5726, Sigma-Aldrich) inhibitors cocktails, according to manufacturer's instructions. Total protein was quantified using Pierce BCA Protein Assay Kit (#23225, Thermo scientific), following the manufacturer protocol. Specific primary antibodies included: mouse monoclonal anti β-Actin antibody (#ab6276; Abcam), rabbit monoclonal anti α-Tubulin (11H10) (#2125, Cell Signaling), rabbit polyclonal anti NFkB p65 (#ab16502, Abcam), rabbit monoclonal anti NF-kB p65 (D14E12) (#8242, Cell Signaling), rabbit monoclonal anti Phospho-NF-kB p65 (Ser536) (93H1) (#3033, Cell Signaling), mouse monoclonal anti IkBα (L35A5) (#4814, Cell Signaling), rabbit monoclonal anti Phospho-IkBα (Ser32) (14D4) (#2859, Cell Signaling), rabbit polyclonal anti Phospho-ERK1/2 (Thr-202/Tyr-204) (#sc-1682, Santa Cruz Biotechnology), rabbit polyclonal anti ERK1/2 (c-14) (#sc-154, Santa Cruz Biotechnology), rabbit polyclonal anti Phospho-AKT1/2/3 (Ser-473) (D9E) (#sc-7985, Santa Cruz Biotechnology), rabbit polyclonal anti AKT1/2/3 (H-136) (#sc-8312, Santa Cruz Biotechnology), rabbit monoclonal anti Vimentin (D21H3) (#5471, Cell Signaling), rabbit monoclonal anti N-Cadherin (D4R1H) (#13116, Cell Signaling), rabbit monoclonal anti β-Catenin (D10A8) (#8480, Cell Signaling), rabbit monoclonal anti Snail (C15D3) (#3879, Cell Signaling), rabbit monoclonal anti Slug (C19G7) (#9585, Cell Signaling), rabbit monoclonal anti E-Cadherin (24E10) (#3195, Cell Signaling), mouse monoclonal anti Nanog (hNanog.2) (#145768-80, eBioscience), mouse monoclonal anti Sox2 (245610) (#MAB2018, R&D systems), mouse monoclonal anti OCT4 (7F9.2) (#MAB4419, Millipore), rabbit monoclonal anti Phospho-Rb (Ser807/811) (D20B12) (#8516, Cell Signaling), rabbit monoclonal anti p21 Waf1/Cip1 (12D1) (#29475, Cell Signaling), rabbit monoclonal anti Cyclin D1 (92G2) (#2978, Cell Signaling), mouse monoclonal anti Cyclin E (HE12) (#05-363, Sigma-Aldrich), rabbit monoclonal anti CDK2 (78B2) (#2546, Cell Signaling), rabbit monoclonal anti CDK4 (D9G3E) (#12790, Cell Signaling), mouse monoclonal anti CDK6 (DCS83) (#3136, Cell Signaling), rabbit monoclonal anti p27 Kip1 (D69C12) (#3686T, Cell Signaling) , mouse monoclonal anti p53 (Bp53-12) (#sc-263, Santa Cruz Biotechnology), mouse monoclonal anti p18 INK4C (DCS118) (#2896, Cell Signaling), rabbit monoclonal anti ERα (D6R2W) (#132585, Cell Signaling), mouse monoclonal anti PR (#KMC912, eBioscience), rabbit monoclonal anti Her2/ErbB2 (#22425, Cell Signaling), rabbit monoclonal anti BCRP/ABCG2 (#ab108312, Abcam), rabbit polyclonal anti hsRANKL (#500-P133, PeproTech), goat polyclonal anti OPG (#AF805, R&D Systems). Horseradish peroxidase-conjugated (HRP) specific secondary antibodies anti-mouse-HRP IgG (#012018, Cell Signaling), anti-rabbit-HRP IgG (#022019, Cell Signaling), and anti-goat-HRP IgG (#sc-2354, Santa Cruz Biotechnology) were used. Proteins were detected using the Novex® ECL HRP chemiluminescent substrate reagent kit (#WP20005, Invitrogen) according to the manufacturer's instructions, and x-ray films developed in a Curix 60 processor (AGFA), or membranes visualized using the Amersham™ Imager 680 (GE Healthcare Life Sciences). Band intensity was calculated using ImageJ software, after background removal, and normalized for β-Actin.

Flow cytometry

For RANK expression analysis, trypsinized cells were washed and incubated with specific mouse antibody against human RANK (#M331, Amgen Inc.) for 30 min at 4°C in the dark, then washed and labelled with 1:100 Cy5 conjugated AffiniPure goat anti-mouse IgG (#115-175-205, Dianova) for 30 min at 4°C, in the dark. For ABACB1/MDR1 expression analysis, trypsinized cells were washed and incubated with specific biotinylated mouse antibody against human CD243 (#348602, Milteny Biotec) for 10 min at 4°C in the dark, then washed and labelled with anti-biotin-PE antibody (#130-111-068, Miltenyi Biotech) for 30 min at 4°C, in the dark. After staining, labelled cells were centrifuged at 120 g, washed, resuspended in 1XPBS with 0.1%FBS (FACS buffer) and acquired on a BD LSRFortessa (BD Biosciences, California, USA). For cell cycle analysis 2–4 × 10^5 cells were seeded in 60 mm dishes under standard conditions for 24 h (asynchronous), synchronized by 24 h serum starvation in low-serum medium (0.1% FBS), and released from serum starvation for another 24 h. At each time point cells were collected, fixed in ice-cold 66% ethanol and kept at 4°C until analysis. Cell cycle analysis was performed using the Propidium iodide (PI) flow cytometry kit (#ab139418, Abcam), following the manufacturer's protocol; and a BD LSRFortessa flow cytometer (BD Biosciences). Analysis was made using FlowJo V10 software.

ELISA assays

For sRANKL quantification, conditioned media was collected from under confluent cells cultured under standard conditions, centrifuged at 4°C for debris removal, and concentrated using Amicon Ultra 30K centrifugal filters (#UFC803024, Millipore). sRANKL was quantified by sandwich ELISA, using 1 μg/ml polyclonal rabbit anti-human sRANKL antibody (#500-P133, PreproTech) as capture antibody, 0.5 μg/ml biotinylated rabbit anti-human sRANKL antibody (#500-P133BT, PreproTech)

as detection antibody, and ABTS (2,2′-Azinobis [3-ethylbenzothiazoline-6-sulfonic acid]-diammonium salt) as substrate (#900-K00, PreproTech). RANKL (#11000457, Amgen Inc.) was used as standard; and protein concentration was normalized for media concentration factor and number of cells.

TRAcP 5b was quantified in mouse serum using the MouseTRAP (TRAcP 5b) ELISA kit (#SB-TR103, IDS), according to the manufacturer instructions.

Tumorsphere formation assay

Adherent non-confluent cells were harvested, washed in 3D Tumorsphere Medium XF (#C-39670, PromoCell), and 10,000 cells/ml seeded in ultra-low attachment 6-well plates. After 7 days, average tumorsphere area was calculated by measuring all tumorspheres > 50 μm in diameter per well. Sphere Forming Capacity (SFC) (%) was determined as the number of mammospheres > 50 μm/number of cells seeded) × 100. 3D tumor spheroid invasion assay was performed as previously described [40], using matrigel (#354230, Corning) and human collagen solution (#04802, StemCell Technologies) (1:1) as matrix, and 2.5 μg/ml RANKL (#11000457, Amgen Inc.).

Viability assays

Cells were seeded in 96 well-plates (2–5 × 10^4 cells/mL), with or without paclitaxel (#Y0000698, Sigma-Aldrich), doxorubicin hydrochloride (#D2975000, Sigma-Aldrich), tamoxifen (#HT904, Sigma-Aldrich), or fulvestrant (#S1191, Selleckchem). Medium was replaced every two days. After 72 h for paclitaxel and doxorubicin, and seven days for tamoxifen and fulvestrant, 1:10 Alamar blue (#DAL1100, Invitrogen) was added to each well and fluorescence was measured 2 hours after incubation (excitation 560 nm; emission 590 nm) in an Infinite M200 microplate reader (Tecan).

To assess the proliferative effect of estrogen, 10,000 cells/well were seeded into 24-well plates under standard conditions for 24 h. After medium removal, cells were washed twice with phenol red-free DMEM:F12 medium (# 11039021, Gibco) supplemented with 5% (v/v) charcoal stripped FBS (csFBS, #12676029, Gibco), and 1% (v/v) Penicillin/Streptomycin (10,000 U/mL Penicillin, 10,000 μg/mL Streptomycin, # 15140122, Gibco). Cells were incubated in the same medium for five days, with or without 10 nM β-estradiol (#E2758, Sigma-Aldrich), and viability was measured with Alamar blue, as described above.

Migration assay

Migration of cancer cells was assessed using a 96-well chemotaxis chamber with polycarbonate filters (8 mm pore size) (#106-8, Neuro Probe), as previously described [22]. Briefly, cells were serum-starved for 24 h and stimulated with 2.5 μg/ml RANKL, neutralized or not with 2.5 mg/ml anti-hTRANCE/TNFSF11 antibody (#MAB626, R&D Systems), for 24 h. Cells were fixed with 2%PFA, stained with crystal violet and images acquired using a Leica DM750 bright field microscope, with 40× magnification. Cells were counted using ImageJ.

Mouse models

All animal experiments were reviewed and approved by the Institutional Animal Welfare Body of the Institute of Molecular Medicine, and licensed by the national regulatory agency Direcção Geral de Alimentação e Veterinária (DGAV). In all studies involving animals, mice were handled and euthanized in accordance with approved institutional, national and international guidelines, applying the Principle of the 3Rs. Four week old NOD scid gamma (NSG) mice (Charles River) were supplemented with subcutaneous 0.36 mg, 60-day release 17β-estradiol pellets (#SE-121, Innovative Research of America), inserted with a trochar in the mid-scapular region of the mouse under mild isofluorane anesthesia prior to breast cancer cell injection, unless otherwise stated. For orthotopic xenografts, cells were harvested at the exponential phase of growth and resuspended in 50% phenol-free matrigel solution (#7338015, Corning) (MCF-7 model with estradiol, 2 × 10^5 cells/ml; MCF-7 model without estradiol, 2 × 10^7/ml; T47D adherent cell model, 2.0 × 10^7 cells/ml). Mice were injected unilaterally or bilaterally with 100 µl of cell suspension, directly into the 2nd thoracic or 4th abdominal fat pad by subcutaneous injection at the base of the nipple. For the experimental metastases model NSG mice were inoculated in the tail vein with 2.5 × 10^6 cells/ml. In the RANKL supplementation model, mice were injected subcutaneously with 0.5 mg/KgBW sRANKL (Amgen), every 48 h for 6 weeks. Tumor growth was monitored weekly by luminescence analysis. Mice were injected with 100 µl/10g body weight (BW) XenoLight D-Luciferin - K+ Salt Bioluminescent Substrate (#122799, PerkinElmer) and after 4 min they were anesthetized with 75 mg/KgBW Ketamine+1mg/KgBW Medetomidine. After approximately 6 min luminescence was analyzed in an IVIS Lumina, using Living Image 3.0 software, and mice recovered with 1 mg/KgBW Atipamezole. Mice were sacrificed by administration of 0.25 mg/KgBW Sodium Pentobarbital. At necropsy, primary tumors were harvested and sectioned into two fragments for paraffin embedding or snapshot freezing. Organs were harvested and paraffin embedded. For circulating tumor cells (CTC) analysis, venous blood was collected by cardiac puncture before sacrifice, into 1.5 mL centrifuge tubes with 5 µl EDTA 0.5M (pH8.0). Erythrocytes were lysed by incubation with 1× RBC Lysis Buffer Multi-species (#00-4300-54, eBioscience) for 10 min at RT. Cells were washed with FACS buffer and centrifuged for 5 min at 500 g. The supernatant was discarded and cells were resuspended in FACS buffer. Samples were analyzed for GFP and RFP expression in a BD LSRFortessa flow cytometer (BD Biosciences).

Immunohistochemistry

5 μm tissue sections from Formalin-Fixed Paraffin-Embedded (FFPE) samples were stained by immunohistochemistry (IHC) for the detection of Ki67, ER and VWF. Deparaffinization and antigen retrieval was performed in a PT Link Pre-Treatment Module for Tissue Specimens (Dako), using Antigen Retrieval Solution pH9.0 (#S2368, Dako), at 94°C for 20 min. Endogenous peroxidase was blocked with Peroxidase Blocking Solution (#411126, Dako) for 10 min at RT, and total protein was blocked by incubation with Protein Block Solution (#411161, Dako), for 20 min at RT. Slides were incubated for 30 min with rabbit anti-human Ki67 primary antibody (1:100, MIB-1, #IR626, Dako) in Antibody Diluent (#411115, Dako) or rabbit anti-human ERα (RTU, EP-1, #IR084, Dako); and for 60 min with polyclonal rabbit anti-human von Willebrand Factor (VWF) primary antibody (1:200, #A0082, Dako) in Antibody Diluent. Slides were incubated with EnVisionTM Detection System, rabbit/mouse (#411083, Dako), according to manufacturer's instructions, followed by 5 min of incubation with DAB (#411066, Dako). Slides were counterstained with hematoxylin, and visualized in a bright field microscope (LeicaDM750 with a Leica ICC50 HD camera). Ki67 was quantified as the percentage of DAB-stained nuclear area over total nuclear area (hematoxylin-stained nuclei regions), from 5 fields at 400× magnification, using the ImageJ software. Microvessel density (MVD) was determined using VWF IHC. A single microvessel was defined as previously described [40], and microvessels were counted in 4 fields at 200× magnification.

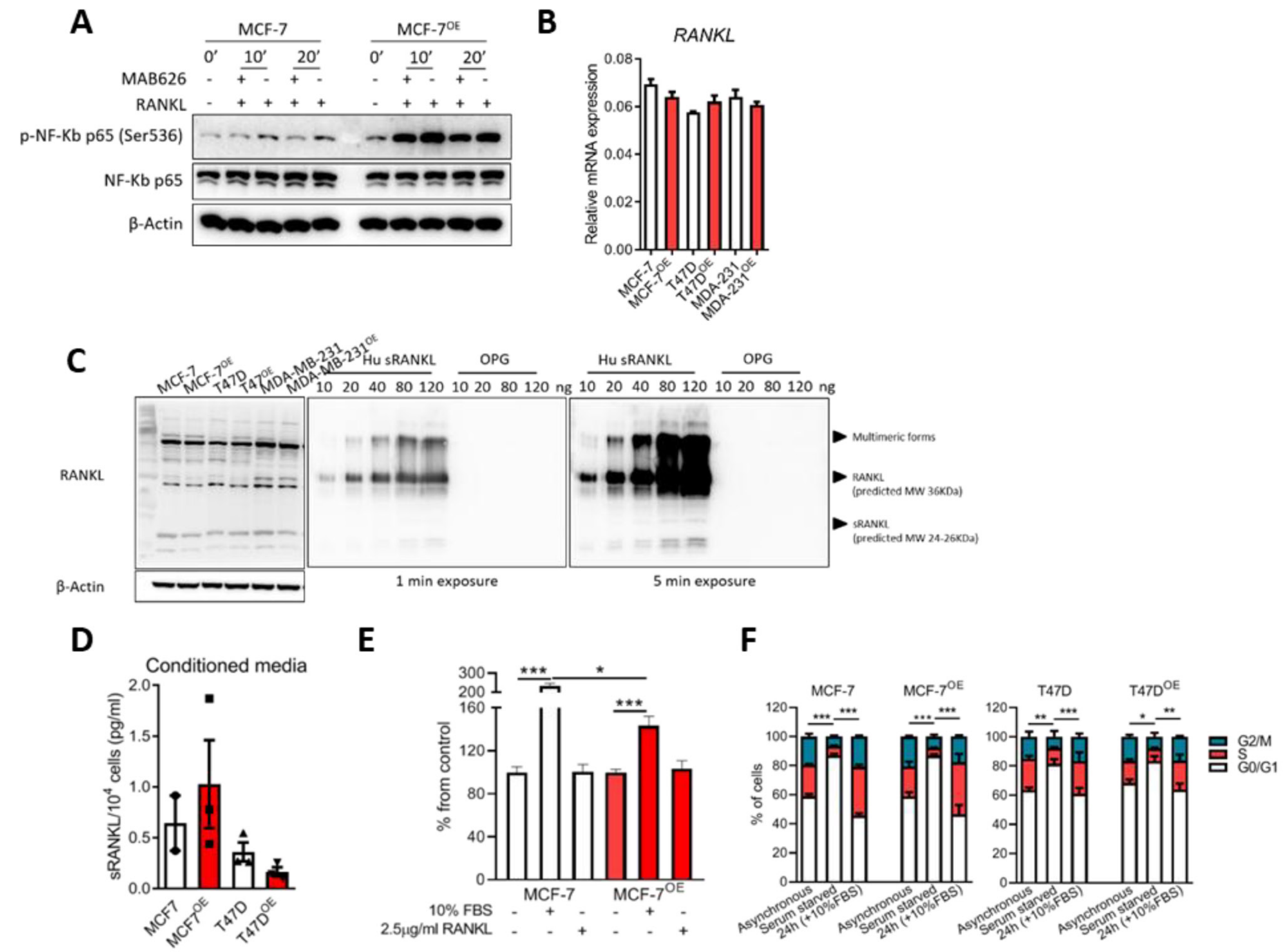

Supplementary Figure 1: (**A**) p65 phosphorylation was analyzed by Western blot after stimuli with RANKL+/-MAB626. (**B**) RT-qPCR of *RANKL* in parental and RANK OE cell lines ($n = 3$). (**C**) Expression of RANKL was analyzed by Western blot using purified RANKL and OPG as control for specific band identification and β-Actin as loading control. (**D**) sRANKL was quantified in cells' conditioned media by ELISA ($n = 3$). (**E**) Cell viability was measured 24 h after release from serum starvation with RANKL or 10%FBS. ($n = 3$) (**F**) Cell cycle analysis by quantification of DNA content with PI staining ($n = 3$).

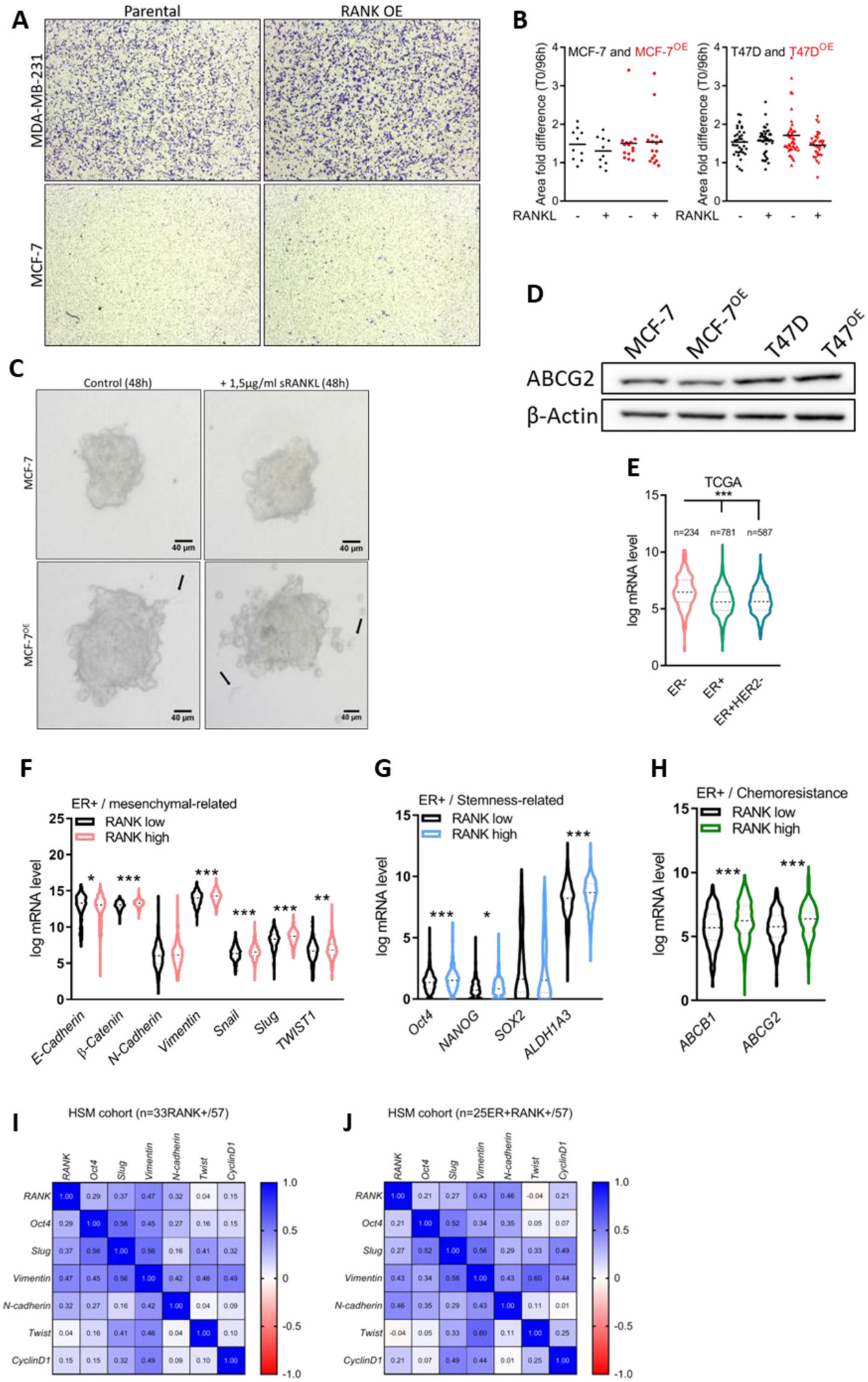

Supplementary Figure 2: Representative images of migration assay and additional characteristics of tumorspheres and ABCG2 expression. (**A**) Representative images of cell migration after 24 h of stimuli with 10%FBS. 40× magnification. (**B**) Tumorspheres' area when cultivated with or without RANKL for 96 h. (**C**) Representative images of tumorspheres embedded in matrigel:collagen matrix and cultivated for 48 h with or without RANKL. (**D**) Western blot analysis of ABCG2 expression with β-Actin as loading control. (**E**) *RANK* expression according to ER status in TCGA (n = 1015) cohort. (**F**, **G**, **H**) Interest gene expression in the ER+ TCGA cohort (n = 781) according to *RANK* expression. (**I**, **J**) Spearman correlation matrixes. Results are presented as the mean ± SEM. $^{*}p < 0.05$, $^{**}p < 0.01$, $^{***}p < 0.001$.

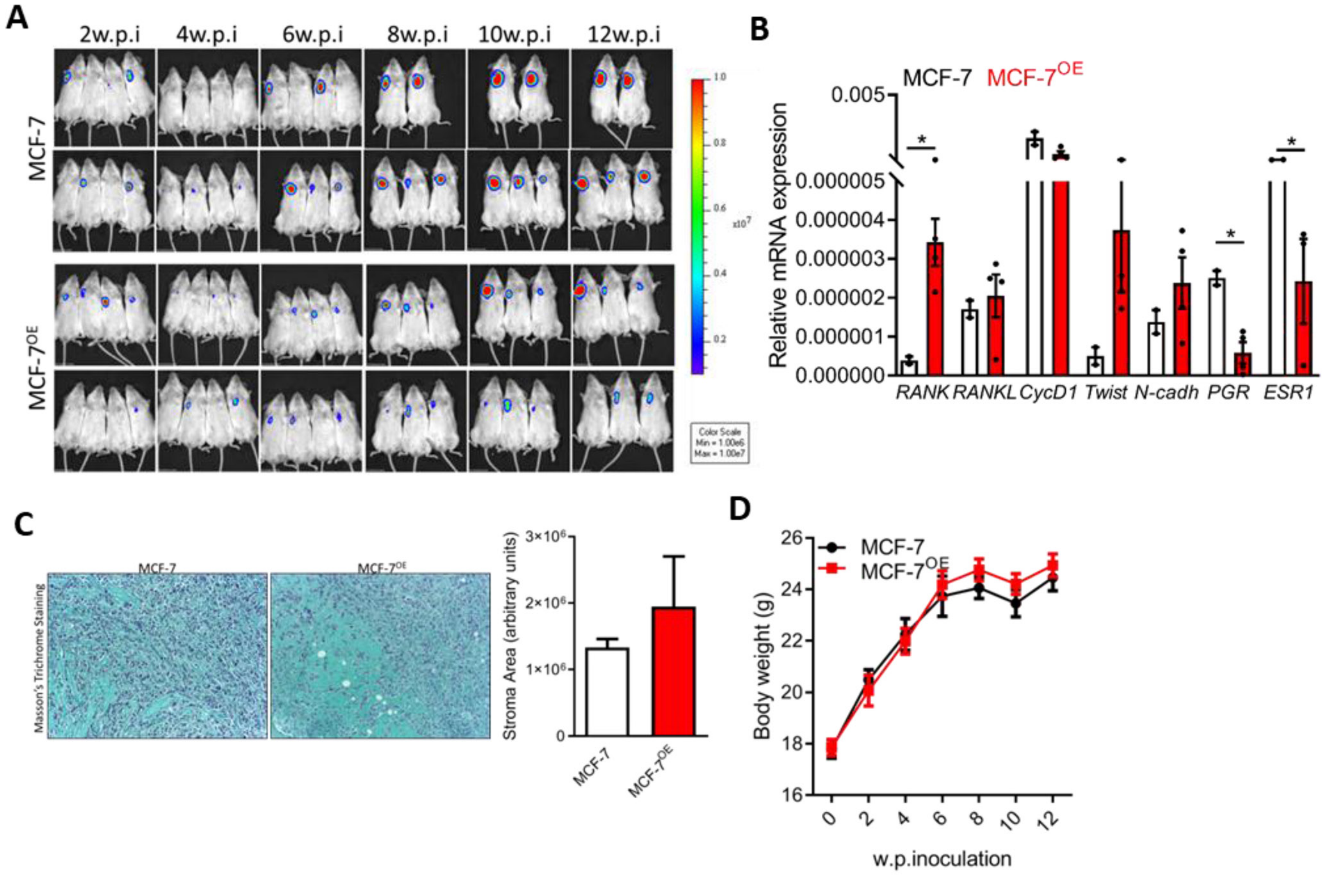

Supplementary Figure 3: RANK OE cells are less proliferative but more invasive *in vivo*. (**A**) BLI analysis of MCF-7 and MCF-7^{OE} xenografts in NSG mice (n = 5–8/group). (**B**) RT-qPCR of indicated genes in tumor tissue. (**C**) Mason's Trichrome staining for quantification of stromal area in mouse xenografts. (**D**) Mouse body weight. Data is presented as mean ± SEM. $^{*}p < 0.05$, $^{**}p < 0.01$, $^{***}p < 0.001$.

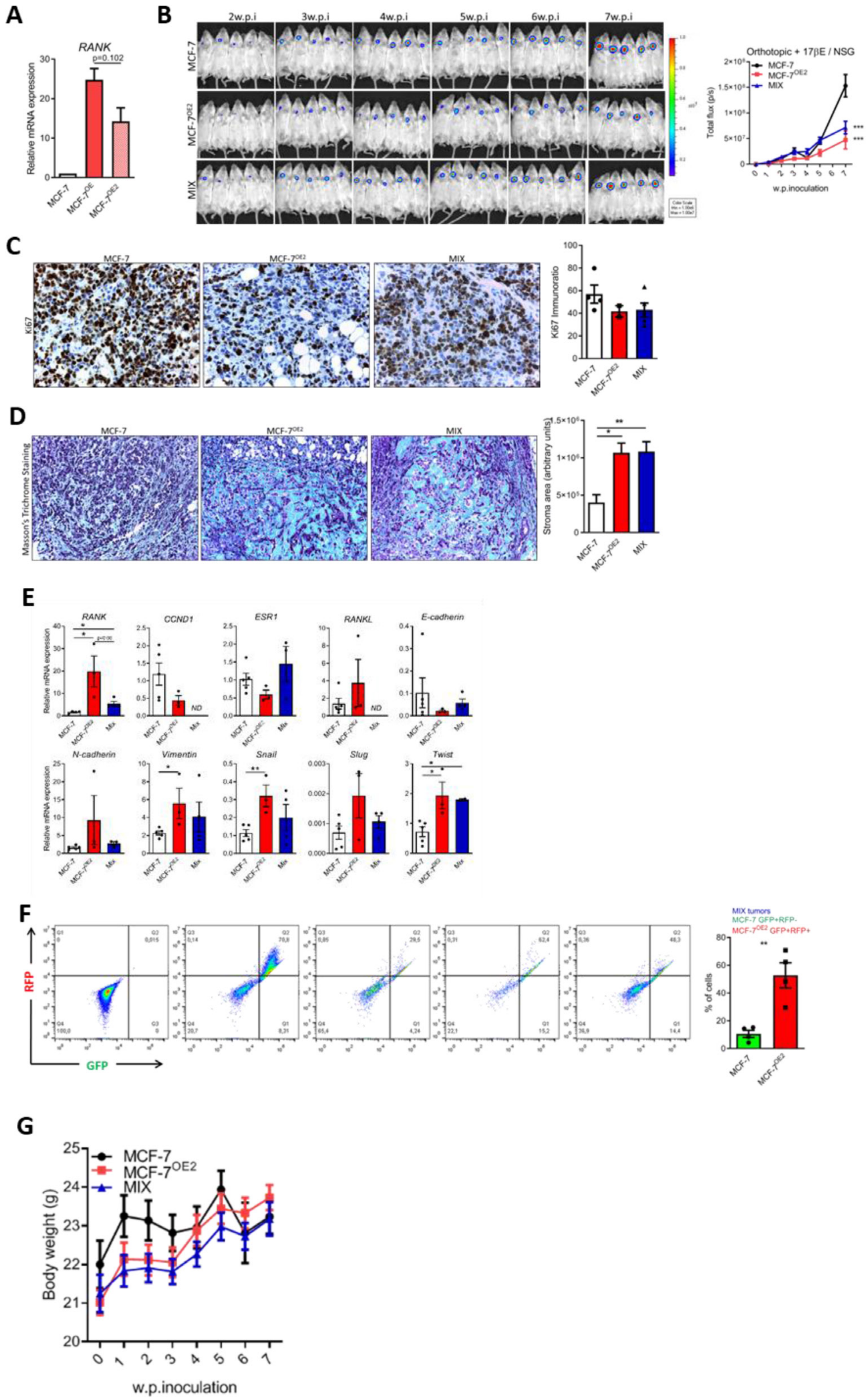

Supplementary Figure 4: A second model of MCF-7 RANK OE orthotopic xenografts (RANK OE2). (**A**) RT-qPCR of RANK in MCF-7, MCF-7^{OE} and MCF-7^{OE2} cell lines (n = 3). (**B**) BLI analysis of MCF-7, MCF-7^{OE2}, or MCF-7 and MCF-7^{OE2} (1:1) (MIX) xenografts in NSG mice (n = 5/group). (**C**) IHC analysis of Ki67. (**D**) Mason's Trichrome staining for quantification of stromal area in mouse xenografts. (**E**) RT-qPCR of indicated genes in tumor tissue. (**F**) Flow cytometry analysis of positive GFP and GFP/RFP cells in MIX tumors at necropsy. (**G**) Mouse body weight.

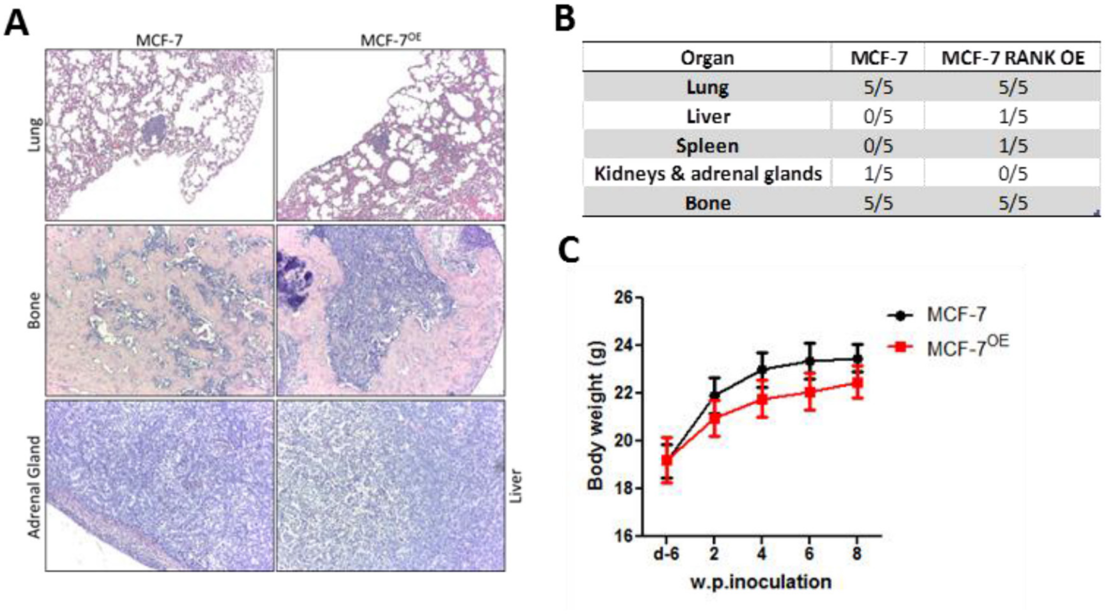

Organ	MCF-7	MCF-7 RANK OE
Lung	5/5	5/5
Liver	0/5	1/5
Spleen	0/5	1/5
Kidneys & adrenal glands	1/5	0/5
Bone	5/5	5/5

Supplementary Figure 5: RANK OE cells are more invasive *in vivo*. (**A**) Representative images of H&E staining of metastases. (**B**) Number of metastases per group. (**C**) Mouse body weight.

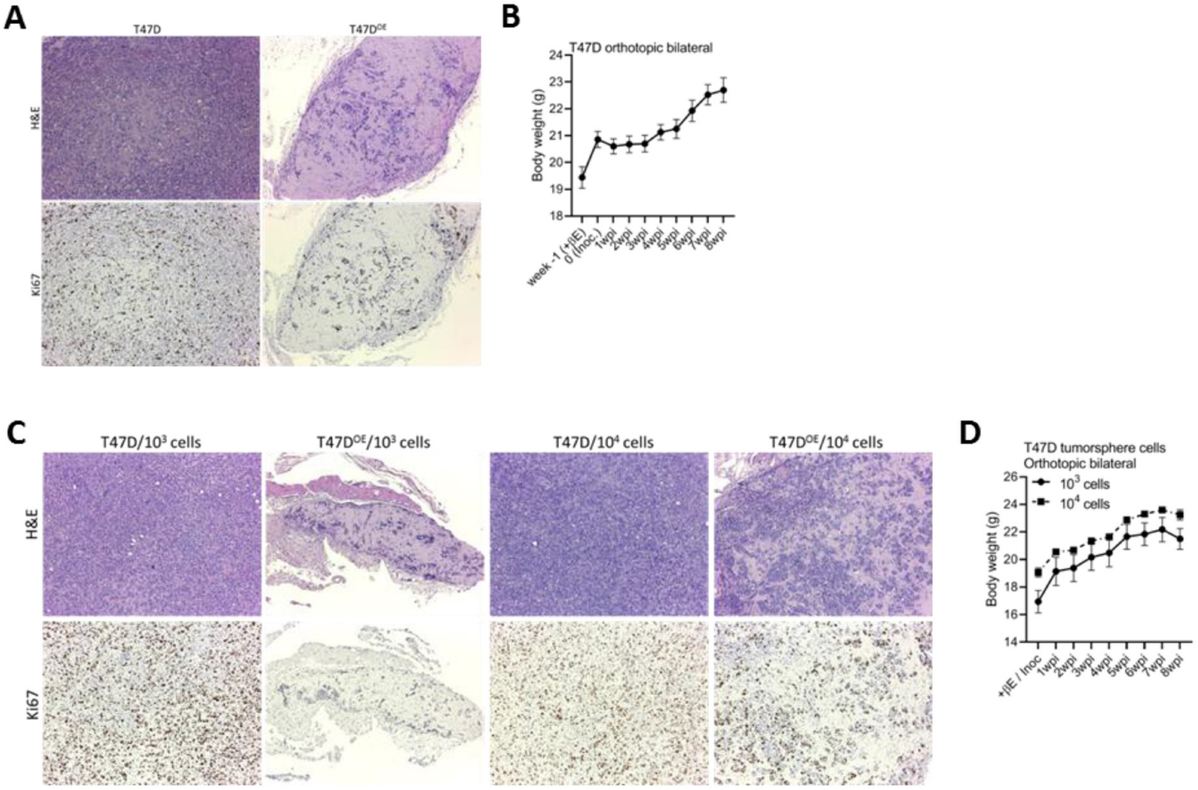

Supplementary Figure 6: T47D xenografts. (**A**) Representative images of H&E staining, and IHC analysis of Ki67 in adherent cell-derived xenografts. (**B**) Mouse body weight. (**C**) Representative images of H&E staining, and IHC analysis of Ki67 in tumorsphere-derived xenografts. (**D**) Mouse body weight.

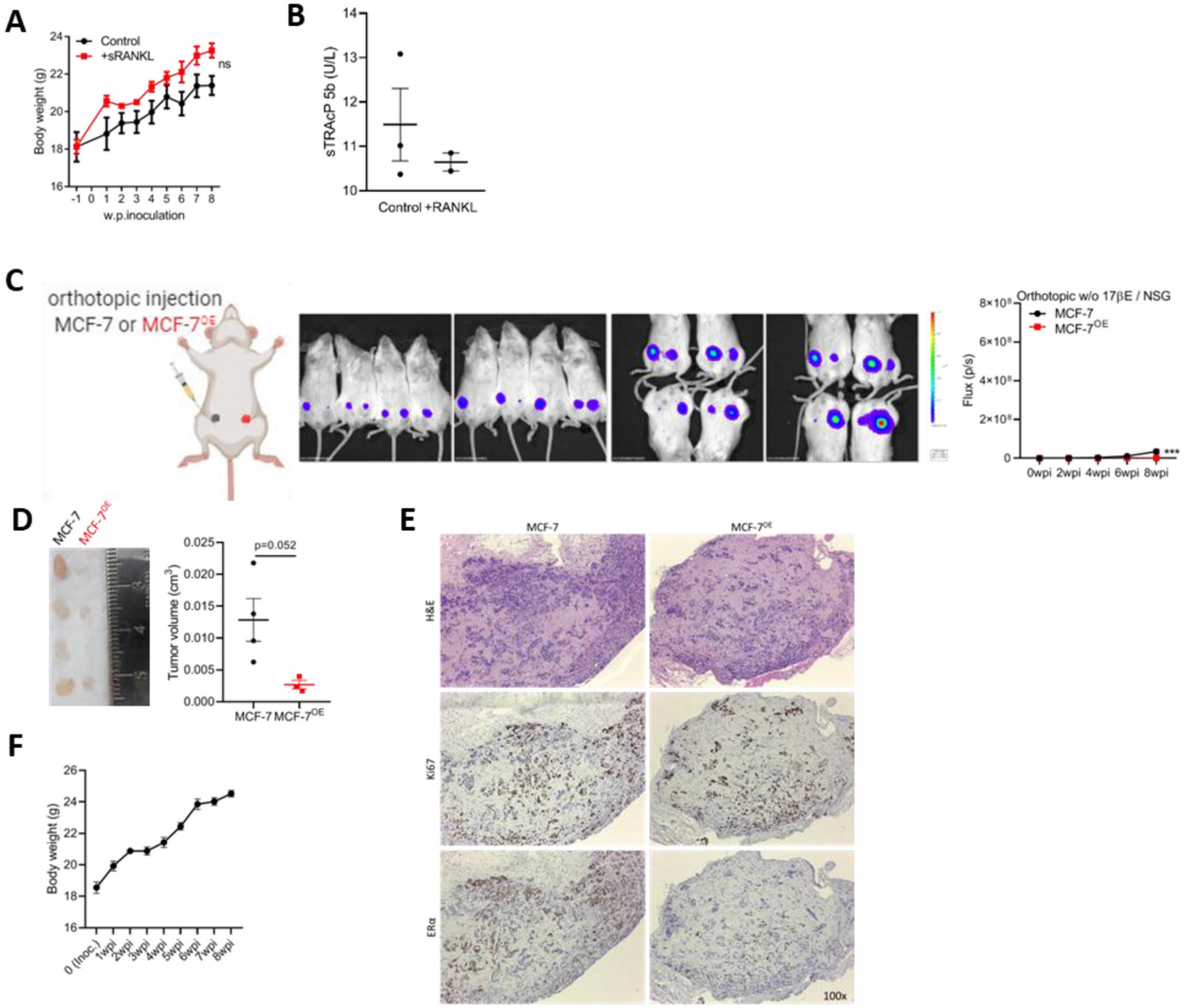

Supplementary Figure 7: MCF-7^{OE} cells are estradiol-dependent. (**A**) Mouse body weight in RANKL supplementation model. (**B**) Osteoclast-specific TRAcp 5b was quantified in the mouse serum by ELISA. (**C**) BLI analysis of MCF-7 and MCF-7^{OE} xenografts in NSG mice without 17βestradiol (17βE) supplementation (n = 4). (**D**) Tumor volume (Tvol= 1/2 (length × width^2)) measured at necropsy. (**E**) Representative images of H&E staining, and IHC analysis of Ki67 and ERα. (**F**) Mouse body weight. Data is presented as mean ± SEM. $^{*}p < 0.05$, $^{**}p < 0.01$, $^{***}p < 0.001$.

Supplementary Table 1: Log2RPKM value of *RANK* (*TNFRSF11A*) gene in each breast cancer cell line based on Cancer Cell Line Encyclopedia (CCLE) database

Cell line	*TNFRSF11A*	ER (+/–)
HCC70_BREAST	2.940109600	–
AU565_BREAST	1.398290561	–
HCC2157_BREAST	1.331681415	–
HCC1143_BREAST	1.197901170	–
HCC1187_BREAST	1.189016004	–
CAL51_BREAST	1.057985114	–
HCC1954_BREAST	0.973932394	–
MDAMB468_BREAST	0.904624149	–
MDAMB231_BREAST	0.838153185	–
MDAMB175VII_BREAST	0.491170875	+
HCC38_BREAST	0.149388964	–
ZR751_BREAST	0.033290241	+
HMEL_BREAST	–0.056751978	–
BT20_BREAST	–0.059324100	–
CAL851_BREAST	–0.233893939	–
HCC1599_BREAST	–0.284225335	–
SKBR3_BREAST	–0.420929371	–
MDAMB415_BREAST	–0.498624298	+
HMC18_BREAST	–0.853397765	–
HCC1937_BREAST	–0.950896290	–
EFM192A_BREAST	–1.258191943	+
HCC1428_BREAST	–1.284746289	+
MDAMB361_BREAST	–1.376981128	+
HCC1419_BREAST	–1.502619572	+
KPL1_BREAST	–1.531007627	+
MCF7_BREAST	–1.557351949	+
HCC202_BREAST	–1.567411078	–
DU4475_BREAST	–1.579180223	–
HCC1806_BREAST	–1.681640483	–
HCC2218_BREAST	–1.705042634	–
T47D_BREAST	–1.793258066	+
UACC812_BREAST	–1.921447176	–
HCC1395_BREAST	–1.973981675	–
MDAMB436_BREAST	–2.104042041	–
CAMA1_BREAST	–2.142396515	+
BT549_BREAST	–2.244616152	–
HS578T_BREAST	–2.246154962	–
BT474_BREAST	–2.382347132	+
HDQP1_BREAST	–2.526858607	–
CAL120_BREAST	–2.715849158	–
MDAMB453_BREAST	–2.779087536	–
HCC1569_BREAST	–2.896640629	–
UACC893_BREAST	–3.557200107	–
JIMT1_BREAST	–3.646321172	+
HS274T_BREAST	–3.653719341	

MDAMB134VI_BREAST	–3.693672779	+
CAL148_BREAST	–4.245191587	–
MDAMB157_BREAST	–4.918962961	–
HS606T_BREAST	–5.098164082	
HS343T_BREAST	–5.374366396	–
HCC1500_BREAST	–5.390897142	–
HS742T_BREAST	–6.161858840	+
HS281T_BREAST	–6.174836524	+
HS739T_BREAST	–6.551758519	
BT483_BREAST	–6.691730693	+
EFM19_BREAST	–7.179649613	+
ZR7530_BREAST	–7.713275303	+

Supplementary Table 2: Clinicopathological characteristics and interest gene expression of the TCGA cohort used in this study. See Supplementary Table 2

Supplementary Table 3: Clinicopathological characteristics according to *RANK* expression[1] in female patients with breast cancer and known ER status from TCGA database

		TCGA BRCA cohort (*n* = 1015)			ER+ cohort (*n* = 781)			ER+HER2- cohort (*n* = 587)		
		RANK[high] (*n* = 507)	RANK[low] (*n* = 508)	*p*-value	RANK[high] (*n* = 390)	RANK[low] (*n* = 391)	*p*-value	RANK[high] (*n* = 293)	RANK[low] (*n* = 294)	*p*-value
**Age at Diagnosis**	**Median**	58	59	0.1412[#]	60	59	0.8418[#]	60	59	0.6229[#]
	**IQR**	48.25–66	49–69		50–68	49–69		50–68	49–68	
**Race**	**Caucasian**	354	340	0.9391[§]	287	270	0.2592[§]	220	213	0.7364[§]
	**Non-caucasian**	117	110		65	76		47	50	
	**NA**	36	58		38	45		26	31	
**Inferred Menopausal Status**	**Pre**	101	111	0.4517[¥]	72	91	0.2626[¥]	58	76	0.1732[¥]
	**Post**	338	331		268	253		204	193	
	**Peri**	20	14		13	12		12	8	
	**NA**	48	52		37	36		19	17	
**Histological sub-type**	**Invasive Ductal**	365	372	0.6732[§]	253	273	0.1476[§]	86	205	0.0975[§]
	**Other**	142	136		137	118		109	89	
**ER status**	**Positive**	341	441	**< 0.0001[§]**	---	---	---	---	---	---
	**Negative**	167	67		---	---	---	---	---	---
**PR status**	**Positive**	296	381	**<0.0001[§]**	327	334	0.5474[§]	245	262	0.0524[§]
	**Negative**	208	125		62	55		47	31	
	**NA**	3	2		1	2		1	1	
**HER2 Status**	**Positive**	71	99	**0.0273[§]**	59	69	0.4360[§]	---	---	---
	**Negative**	389	369		294	293		---	---	---
	**NA**	47	40		37	29		---	---	---
**Tumor Stage**	**1**	88	87	0.7605[¥]	71	69	0.7473[¥]	58	58	0.9368[¥]
	**2**	294	278		219	208		163	156	
	**3**	109	122		88	99		65	71	
	**4**	9	9		8	6		5	5	
	**NA**	7	12		3	9		2	4	
**Neo Therapy**	**Yes**	3	10	0.0902[§]	5	8	0.5758[§]	3	6	0.5044[§]
	**No**	504	497		385	383		290	288	
**N**	**0**	241	240	0.8001[§]	167	180	0.2179[§]	131	142	0.4090[§]
	**+**	263	253		220	198		161	152	
	**Nx**	3	15		3	13		1	8	
**T**	**1**	142	122	0.4638[¥]	115	95	0.3785[¥]	94	7	0.3114[¥]
	**2**	285	299		208	230		148	170	
	**3**	64	64		54	52		42	39	
	**4**	15	20		13	12		9	7	
	**x**	1	3			2			1	
**Overall Survival (days)**	**Median**	396.5	463	0.6072[#]	434	440	0.6262[#]	410	414.5	0.5740[#]
	**IQR**	158.5–1272	170–1173		196.5–1221	169–1209		211–1159	170–1094	
**Survival Status**	**Alive**	459	460	0.9146[§]	359	359	1.0000[§]	282	277	0.2501[§]
	**Deceased**	49	47		31	32		11	17	

[1]Cut-off for dichotomization was the median in each cohort (All cases, logRANK mRNA level = 5.812; ER+, logRANK mRNA level = 5.617; ER+HER2-, logRANK mRNA level = 5.64). [#]Two-tailed unpaired *t*-test; [§]Fisher's exact test; [¥]Chi-square test.

Supplementary Table 4: Mesenchymal, stem cell and chemoresistance related genes are positively correlated with RANK expression in TCGA ER+ and ER+HER2- cohorts

	TNFRSF11A vs.	ER+ (*n* = 781)				ER+HER2- (*n* = 587)			
		**Pearson r**	**95% confidence interval**	**R^2**	***P***	**Pearson r**	**95% confidence interval**	**R^2**	***P***
Epithelial-mesenchymal	***E-Cadherin***	–0.0528	–0.1225 to 0.0174	0.00279	0.1406	–0.0108	–0.0917 to 0.0702	0.00012	0.7937
	β-catenin	0.2678	0.2014 to 0.3317	0.07172	**< 0.0001**	0.2320	0.1540 to 0.3072	0.05384	**< 0.0001**
	Vimentin	0.2242	0.1565 to 0.2898	0.05025	**< 0.0001**	0.2189	0.1405 to 0.2946	0.04793	**< 0.0001**
	N-Cadherin	0.0358	–0.0345 to 0.1057	0.00128	0.3179	0.0683	–0.0127 to 0.1484	0.00466	0.0984
	Snail	0.1570	0.0878 to 0.2247	0.02464	**< 0.0001**	0.1862	0.1069 to 0.2632	0.03468	**< 0.0001**
	Slug	0.2521	0.1852 to 0.3167	0.06356	**< 0.0001**	0.2568	0.1796 to 0.3308	0.06594	**< 0.0001**
	TWIST1	0.1432	0.0738 to 0.2112	0.02051	**< 0.0001**	0.1706	0.0901 to 0.2481	0.02911	**< 0.0001**
Stem cell related	***SOX2***	–0.0760	–0.1456 to –0.0061	0.00581	**0.0330**	–0.0361	–0.1166 to 0.4501	0.00130	0.3833
	NANOG	0.0678	–0.0024 to 0.1373	0.00460	0.0582	0.0483	–0.0328 to 0.1287	0.00233	0.2429
	Oct4	0.1944	0.1259 to 0.2610	0.03778	**< 0.0001**	0.2129	0.1433 to 0.2889	0.04535	**< 0.0001**
Chemoresistance related	***ALDH1A3***	0.2265	0.1589 to 0.2920	0.05130	**< 0.0001**	0.2503	0.1729to 0.3247	0.06267	**< 0.0001**
	ABCB1	0.2248	0.1572 to 0.2904	0.05055	**< 0.0001**	0.2220	0.1436 to 0.2976	0.04928	**< 0.0001**
	ABCG2	0.2228	0.1551 to 0.2884	0.04964	**< 0.0001**	0.1934	0.1142 to 0.2701	0.03739	**< 0.0001**

Supplementary Table 5: Clinicopathological characteristics of patients included in HSM cohort

		HSM cohort (*n* = 57)
**Age at Diagnosis**	**Median**	60.36
	**IQR**	50.37–79.21
**Tumor grade**	**1**	5 (8.8%)
	**2**	37 (64.9%)
	**3**	13 (22.8%)
	**NA**	2 (3.5%)
**Histological sub-type**	**Invasive carcinoma**	53 (92.9%)
	**Other**	4 (7.1%)
**ER status**	**Positive**	43 (75.4%)
	**Negative**	14 (24.6%)
**PR status**	**Positive**	40 (70.2%)
	**Negative**	17 (29.8%)
**HER2 Status**	**Positive**	10 (17.5%)
	**Negative**	46 (80.7%)
	**NA**	1 (1.8%)
**T**	**1**	15 (26.3%)
	**2**	32 (56.1%)
	**3**	8 (14.0%)
	**4**	2 (3.5%)
**N**	**0**	26 (45.6%)
	**+**	31 (54.4%)
**RANK expression (mRNA)**	**Positive**	33 (57.9%)
	**Negative**	24 (42.1%)
**Metastatic disease**	**Yes**	20 (35.1%)
	**No**	37 (64.9%)
**Relapse-free survival (days)**	**Median**	1054
	**IQR**	333.5–1954
**Follow-up (days)**	**Median**	2086
	**IQR**	1363–2400
**Survival Status**	**Alive**	38
	**Deceased**	19
**Overall Survival (days)**	**Median**	1351
	**IQR**	850.5–1947
